# Bladder Cancer‐Derived Small Extracellular Vesicles Promote Tumor Angiogenesis by Inducing HBP‐Related Metabolic Reprogramming and SerRS O‐GlcNAcylation in Endothelial Cells

**DOI:** 10.1002/advs.202202993

**Published:** 2022-08-31

**Authors:** Xinyuan Li, Xiang Peng, Chunlin Zhang, Xuesong Bai, Yang Li, Guo Chen, Huixia Guo, Weiyang He, Xiang Zhou, Xin Gou

**Affiliations:** ^1^ Department of Urology The First Affiliated Hospital of Chongqing Medical University Chongqing 400016 China; ^2^ Centre for Excellence in Molecular Cell Science Shanghai Institute of Biochemistry and Cell Biology Chinese Academy of Sciences Shanghai 200031 China; ^3^ Chongqing Key Laboratory of Molecular Oncology and Epigenetics The First Affiliated Hospital of Chongqing Medical University Chongqing 400016 China

**Keywords:** angiogenesis, glutamine‐fructose‐6‐phosphate aminotransferase 1, metabolic reprogramming, O‐GlcNAcylation, small extracellular vesicles

## Abstract

A malformed tumour vascular network provokes the nutrient‐deprived tumour microenvironment (TME), which conversely activates endothelial cell (EC) functions and stimulates neovascularization. Emerging evidence suggests that the flexible metabolic adaptability of tumour cells helps to establish a metabolic symbiosis among various cell subpopulations in the fluctuating TME. In this study, the authors propose a novel metabolic link between bladder cancer (BCa) cells and ECs in the nutrient‐scarce TME, in which BCa‐secreted glutamine‐fructose‐6‐phosphate aminotransferase 1 (GFAT1) via small extracellular vesicles (sEVs) reprograms glucose metabolism by increasing hexosamine biosynthesis pathway flux in ECs and thus enhances O‐GlcNAcylation. Moreover, seryl‐tRNA synthetase (SerRS) O‐GlcNAcylation at serine 101 in ECs promotes its degradation by ubiquitination and impeded importin *α*5‐mediated nuclear translocation. Intranuclear SerRS attenuates vascular endothelial growth factor transcription by competitively binding to the GC‐rich region of the proximal promotor. Additionally, GFAT1 knockout in tumour cells blocks SerRS O‐GlcNAcylation in ECs and attenuates angiogenesis both in vitro and in vivo. However, administration of GFAT1‐overexpressing BCa cells‐derived sEVs increase the angiogenetic activity in the ECs of GFAT1‐knockout mice. In summary, this study suggests that inhibiting sEV‐mediated GFAT1 secretion from BCa cells and targeting SerRS O‐GlcNAcylation in ECs may serve as novel strategies for BCa antiangiogenetic therapy.

## Graphical abstract

The starving intratumor microenvironment facilitates the secretion of bladder cancer (BCa)‐derived small extracellular vesicle containing glutamine‐fructose‐6‐phosphate aminotransferase 1 (a key enzyme in hexosamine biosynthesis pathway [HBP]), which forge a metabolic link between BCa cells and endothelial cells. Increased HBP flux in endothelial cells improves angiogenetic activities by enhancing SerRS O‐GlcNAcylation.

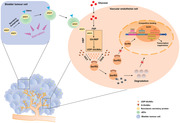

## 1 Introduction

Bladder cancer (BCa) is one of the most prevalent malignancies with the characteristics of strong vascularization.^[^ ^1^, ^2^ ^]^ However, the tumor vascular network is usually malformed because of compression from rapidly growing cancer cells, causing inadequate perfusion and deficiencies in the levels of nutrients and oxygen.^[^ ^3^, ^4^ ^]^ Conversely, starvation and hypoxia strongly drive the activation of endothelial cell (EC) functions, provoke continuous neovascularization, and further contribute to cancer progression.^[^ ^5^, ^6^ ^]^ Therefore, identifying novel and viable antiangiogenetic targets, with the consideration of the stressed intratumor environment, seems to be promising for BCa therapy.

Cancer metabolic reprogramming, as an essential feature of tumorigenesis, not only satisfies the bioenergetic, biosynthetic, and redox requirements for the rapid proliferation of tumor cells^[^ ^7^, ^8^, ^9^ ^]^ but also empowers cancer cells with the metabolic adaptability to flexibly coordinate different cellular functions, respond to various external stimulations from the fluctuating tumor microenvironment (TME), and establish a metabolic symbiosis among various cell subpopulations to reshape the TME.^[^ ^10^, ^11^, ^12^, ^13^ ^]^ Therefore, gaining further insights into the connection between the metabolic reprogramming of BCa cells and that of ECs, especially in the nutrient‐scarce environment, will help to reveal more molecular mechanisms of aberrant tumor angiogenesis and identify more intriguing and underexplored therapeutic targets for BCa antiangiogenetic treatment.

Glucose metabolism, including through glycolysis, the pentose phosphate pathway and glycogenesis, produces the major energy for cell functions, and supplies precursors for biomacromolecule synthesis.^[^ ^14^, ^15^ ^]^ Enhanced aerobic glycolysis, that is, the “Warburg effect,” is a general hallmark of cancer cells and the main energic source of ECs in the tumor vasculature, as evidenced by increased expression levels of glycolytic enzymes.^[^ ^16^, ^17^, ^18^, ^19^ ^]^ The hexosamine biosynthetic pathway (HBP), branching off from the glycolysis pathway, not only results in the synthesis of uridine diphosphate N‐acetylglucosamine (UDP‐GlcNAc) for O‐linked *β*‐N‐acetylglucosamine (O‐GlcNAc) protein modification (O‐GlcNAcylation) and glycoconjugate biosynthesis but also in the maintenance of the homeostatic balance of glucose and glutamine metabolism.^[^ ^20^, ^21^, ^22^ ^]^ Currently, accumulating evidence delineates that the HBP flux and protein O‐GlcNAc levels in cancer cells are prominently increased, especially under the condition of nutrient scarcity.^[^ ^23^, ^24^ ^]^ Nevertheless, the role of HBP‐related metabolic reprogramming and O‐GlcNAcylation in ECs remains unclear, as does the question of whether there is metabolic symbiosis between BCa cells and ECs.

Glutamine fructose‐6‐phosphate amidotransferase (GFAT), as the first and rate‐limiting enzyme in the HBP, utilizes glutamine to catalyze the conversion of fructose‐6‐phosphate (F6P) to glucosamine‐6‐phosphate (GlucN6P) and UDP‐GlcNAc.^[^ ^25^, ^26^ ^]^ Kim et al. demonstrated that genetic ablation of GFAT1 completely attenuated proliferation and promoted death of pancreatic ductal adenocarcinoma cells.^[^ ^27^ ^]^ Sharma and her colleagues also elucidated that suppressing GFAT1 expression with 6‐diazo‐5‐oxo‐l‐norleucine (DON) strikingly diminished the potential for self‐renewal in and the metastatic capacity of pancreatic cancer cells.^[^ ^22^ ^]^ In addition, high GFAT1 expression was identified as an independent predictor of adverse clinical outcomes, such as poor overall survival and recurrence‐free survival, in pancreatic cancer^[^ ^28^ ^]^ and hepatocellular carcinoma patients.^[^ ^29^ ^]^ Intriguingly, GFAT1 was shown to be secreted via B‐cell‐derived exosomes as a nonclassic secretory protein (NSP) without a signal peptide.^[^ ^30^ ^]^ Our previous study demonstrated that the strengthened secretory autophagy observed in a nutrient‐deprived environment promotes NSP secretion via LC3‐conjugated small extracellular vesicles.^[^ ^31^ ^]^ We, therefore, speculate that BCa‐derived GFAT1, through intercellular communication in the TME, mediates HBP‐related metabolic reprogramming and functional regulation in ECs.

Small extracellular vesicles (sEVs) are cell‐secreted extracellular membranous vesicles ranging in size from 30 to 200 nm in diameter, including exosomes, microvesicles, and other membranous particles.^[^ ^32^, ^33^ ^]^ An increasing number of studies have documented that sEVs deliver diverse cargos, including proteins, lipids, and various nucleic acids, facilitating intercellular signal transduction.^[^ ^34^, ^35^ ^]^ More recently, tumor‐derived sEVs have been increasingly identified as mediators of a novel mechanism that promotes tumor angiogenesis.^[^ ^36^ ^]^ For example, the Ras/syntenin‐1 axis triggered the release of sEVs loaded with miR‐494‐3p, which promoted lung cancer cell migration and angiogenesis.^[^ ^37^ ^]^ Interleukin‐35 altered the mRNA profiles of breast cancer cell‐derived sEVs and facilitated angiogenesis by activating the Ras/ERK signaling pathway.^[^ ^38^ ^]^ In addition, Huang et al. revealed that perivascular cell‐derived sEVs containing Gas6 elicited the recruitment of endothelial progenitor cells by activating the Axl pathway and augmented tumor revascularization.^[^ ^39^ ^]^ However, the role and potential mechanism of BCa‐derived sEVs in regulating the metabolism and function of ECs, especially in the nutrient‐scarce intratumor microenvironment, remain poorly understood.

Seryl‐tRNA synthetase (SerRS) is a well‐known member of the aminoacyl‐tRNA synthetase family with the classic role of charging serine onto the cognate tRNA for protein synthesis.^[^ ^40^ ^]^ Recent studies documented a specific function of SerRS in impeding angiogenesis, distinguishing it from other tRNA synthetases.^[^ ^41^ ^]^ In this regard, SerRS was translocated to the nucleus due to its vertebrate‐specific, carboxyl‐terminal domain (UNE‐S domain), where vascular endothelial growth Factor A (VEGFA) expression was increased by transcriptional repression and the recruitment of histone deacetylase NAD‐dependent protein deacetylase sirtuin‐2 (SIRT2). Interestingly, Zhao et al. recently found that intracellular glucose and glutamine metabolism strikingly increased the nuclear translocation of SerRS by facilitating its acetylation at lysine 323 and further rewiring lipid metabolism in mammary gland epithelial cells.^[^ ^42^ ^]^ In addition, the anti‐angiogenetic role of SerRS is inactivated through phosphorylation by ataxia telangiectasia mutated (ATM) and ATM and RAD3‐related (ATR) under hypoxia and starvation.^[^ ^43^ ^]^ Therefore, we deduce that HBP‐related metabolic rewiring plausibly mediates BCa neovascularization by regulating SerRS in ECs.

In this study, we explored the effect of bladder tumor sEV‐derived GFAT1 on metabolic reprogramming in ECs and angiogenetic regulation under nutrient‐scarce conditions. We propose a novel mechanism by which BCa‐secreted GFAT1 delivered by sEVs augments HBP flux and O‐GlcNAcylation levels in ECs and are the first illuminate the functional importance of SerRS O‐GlcNAcylation in BCa neovascularization. More importantly, our findings reveal a relationship between HBP‐related metabolic rewiring and symbiosis among different cell populations in the TME, which suggests promising therapeutic targets and opens up a novel perspective on antiangiogenic treatment for BCa.

## 2 Results

### 2.1 HBP Fluxes are Prominently Increased in BCa‐Isolated ECs

**Figure 1:**
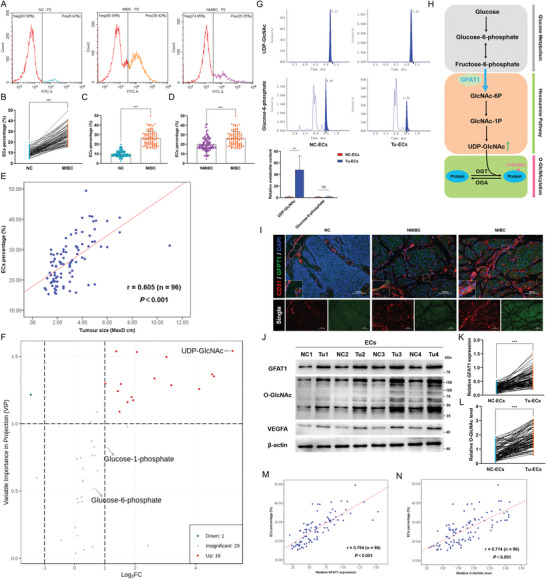
GFAT1 reprograms glucose metabolism by increasing HBP flux in ECs and thus enhances protein O‐GlcNAcylation. A) The EC percentage was detected in paired tumor tissues (Tu) and normal urothelium (NC) of MIBC patients (*n* = 96) and Tu of NMIBC patients (*n* = 124) by flow cytometry. B–D) The EC percentage was compared by unpaired and paired 2‐tailed Student's *t*‐test. ***p < 0.001, **p < 0.01. E) The correlation between MIBC tumor sizes (*n* = 96) and EC percentage. *p*‐Value was generated from Pearson's correlation coefficient (*r*). F) Volcano plot showed all detected glucose metabolism‐related metabolites in ECs isolated from NC (NC‐ECs, *n* = 5) and Tu (Tu‐ECs, *n* = 5). The red dots represent the significantly up‐regulated metabolites in the Tu‐ECs; the green dot represents the remarkably down‐regulated metabolites, and the gray dots indicate no significant difference. G) The relative levels of UDP‐GlcNAc and Glucose‐6‐phosphate in paired NC‐ECs and Tu‐ECs (*n* = 20) (*top*), and quantitative analysis (*down*). **p < 0.01, ns represents no significant difference. Metabolite content was normalized according to the level in NC‐ECs. H) Schematic representation of HBP in the glucose metabolism process and HBP‐mediated O‐GlcNAcylation. Glucose feed into HBP which produces UDP‐GlcNAc for O‐GlcNAcylation. GFAT1 is the switch and rate‐limiting enzyme of HBP. I) IF assays detected the expression of GFAT1 (green), and the number of vessel (marked by CD31, red) in paired Tu slices and NC slices of MIBC patients (*n* = 96) and Tu slices of NMIBC patients (*n* = 124). Scale bar: 100 µm. J) IB assays examined the expression of GFAT1, O‐GlcNAc, SerRS, and VEGFA in paired Tu‐ECs and NC‐ECs of MIBC patients. Relative GFAT1 expression levels (K) and O‐GlcNAc levels (L) were compared between the NC‐ECs and Tu‐ECs from 96 MIBC patients. ***p < 0.001. M) Correlation analysis between GFAT1 expression in Tu‐ECs and EC percentage in Tu (*n* = 96). N) Correlation analysis between O‐GlcNAc levels in Tu‐ECs and EC percentage in Tu (*n* = 96).

To explore the correlation between tumor angiogenesis and clinicopathological characteristics, we first detected the EC percentage in paired normal bladder urothelium and tumor tissues from 96 MIBC patients and the single tumor tissues of 124 NMIBC patients (**Table** **1**). The results of flow cytometry showed that the EC percentage in the tumor tissues (Tu) of MIBC patients was strikingly higher than that in the paired normal urothelium tissues (NC) and the Tu of NMIBC patients (**Figure** **1**A–D; Figure S1A, Supporting Information). Additionally, Pearson correlation analysis illuminated a positive correlation between EC percentage and tumor size (*r* = 0.605, *p* < 0.001, *n* = 96) (Figure 1E). Next, we isolated and purified ECs from paired tissues of MIBC patients and utilized metabonomic profiling and targeted analysis to reveal the features of glucose metabolism, which provides most of the energy used in the tumor vasculature.^[^ ^44^, ^45^ ^]^ As illustrated in Figure 1F and Figure S1B, Supporting Information, there were 16 metabolites with significantly higher levels in Tu‐derived ECs (Tu‐ECs) than in NC‐isolated ECs (NC‐ECs), of which the differences in levels of UDP‐GlcNAc, the end‐product of the HBP, and the key donor substrate for O‐GlcNAcylation, were the most prominent. Therefore, we speculate that the HBP flux in Tu‐ECs was increasingly branched off from the glycolysis pathway, another embranchment of glucose metabolism. Interestingly, through further metabolite detection in more EC samples, we found that, in contrast to UDP‐GlcNAc levels, the levels of some metabolites before the HBP branch (glucose‐6‐phosphate and glucose‐1‐phosphate) were not different between NC‐ECs and Tu‐ECs (Figure 1G; Figure S1C, Supporting Information). These findings raise the intriguing possibility that GFAT1, as the switch and the crucial rate‐limiting enzyme in the HBP, probably reprograms the glucose metabolic process by accelerating the HBP flux in Tu‐ECs (Figure 1H).

**Table 1:** The clinicopathologic factors of patients with NMIBC and MIBC

Parameter	NMIBC	MIBC	*p‐*Value
Number	124	96	
Gender			0.374
Male	99	82	
Female	25	14	
Age (year)			1.000
<65	79	62	
≥65	45	34	
Tumor size (MaxD cm)			**<0.001**
<2	82	37	
≥2	42	59	
Differentiation grade			0.078
Well	67	40	
Poor	57	56	
Nidus			0.831
Single	111	85	
Multiple	13	11	
T stage			—
T1	124	0	
T2	0	96	
Lymph node status			—
Negative	124	96	
Positive	0	0	
Distant metastasis site			—
Negative	124	96	
Positive	0	0	

MaxD, maximum diameter.

The bold number represents the *p*‐value with significant difference.

### 2.2 GFAT1 Promotes Tumor Angiogenesis by Strengthening HBP‐Mediated O‐GlcNAcylation in Tu‐ECs

Next, we attempted to explore whether GFAT1 levels are accordingly increased in Tu‐ECs and whether it plays a crucial role in angiogenesis. The results of immunofluorescence (IF) assays (Figure 1I) and immunohistochemical (IHC) assays of serial sections (Figure S1D, Supporting Information) suggested that the expression levels of GFAT1 and VEGFA were both higher in tumor cells and ECs in Tu than those in NC, consistent with the richer vascular distribution (marked by CD31) in Tu, especially in MIBC. Accordingly, we used IB analyses to detect that the expression levels of GFAT1 and VEGFA in the Tu‐ECs were higher to various degrees than those of the NC‐ECs (Figure 1J,K). In addition, we found notable increases in the O‐GlcNAc level in Tu‐ECs, indicating that HBP‐mediated O‐GlcNAcylation is synergistically increased with increases in GFAT1 levels in Tu‐ECs (Figure 1J,L). The results of correlation analyses illuminated that the higher levels of GFAT1 and O‐GlcNAc in the Tu‐ECs were both markedly correlated with a larger EC percentage (Figure 1M,N). On the basis of these findings, we deduce that GFAT1 promotes a glucose metabolism shift to the HBP branch, which augments O‐GlcNAcylation in Tu‐ECs, eventually leading to the induction of angiogenesis.

### 2.3 GFAT1 is Enriched in BCa‐Derived sEVs and Modulates Tumor Angiogenesis

**Figure 2:**
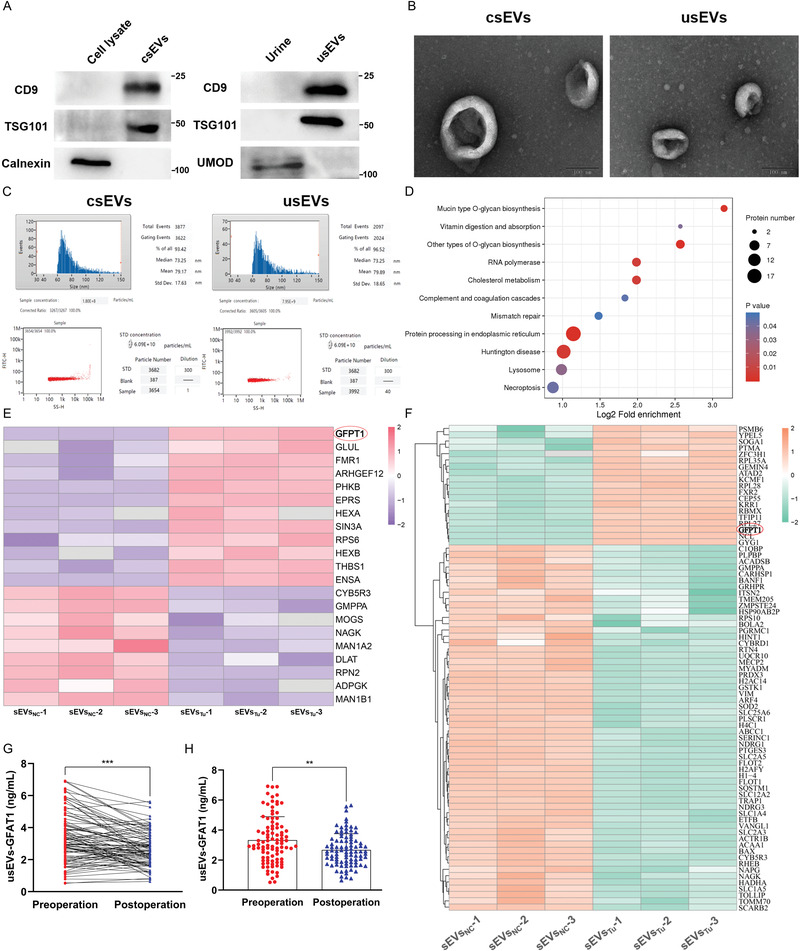
BCa‐derived sEVs‐GFAT1 modulates tumor angiogenesis. A) IB illustrating the expression of four categories of sEV markers (sEVs derived from cell medium [csEVs]: CD9, TSG101, and calnexin; usEVs: CD9, TSG101, and UMOD). B) sEV morphologies were observed by TEM. Scale bar: 100 nm. C) NTA and Flow NanoAnalyzer showing the particle size ranges (top) and concentrations (down) of csEVs and usEVs. D) Gene Ontology analysis of identified upregulated proteins in the sEVs derived from BCa cell medium (sEVs_Tu_). Cluster heat map illustrating the differentially expressed glucose metabolism‐related proteins (E) and nonclassic secretory proteins (F) between sEVs_NC_ (*n* = 3) and sEVs_Tu_ (*n* = 3). Proteins with fold variation greater than 2 are shown. G,H) The concentration of GFAT1 in the pre‐usEVs and post‐usEVs of the same MIBC patients was detected by ELISA and quantitatively analyzed by unpaired and paired 2‐tailed Student's *t*‐test (pre‐usEVs: *n* = 96, post‐usEVs: *n* = 92). ***p < 0.001, **p < 0.01.

With this deduction in mind, the focus turned toward exploring the mechanism of GFAT1 action in ECs. GFAT1, as a type of NSP, has been reported to be secreted via exosomes,^[^ ^30^ ^]^ consistent with our previous finding that secretory autophagy promoted sEV‐mediated NSP secretion in the TME.^[^ ^31^ ^]^ Therefore, we isolated sEVs from the medium of normal urothelial cells (sEVs_NC_) and BCa cells (sEVs_Tu_) (**Figure** **2**A–C), and examined the protein profiles of sEVs_NC_ and sEVs_Tu_ using 4D‐label‐free LC–MS/MS (Figure S2A, Supporting Information). As expected, the Gene Ontology (GO) and Kyoto Encyclopedia of Genes and Genomes (KEGG) analyses suggested that the upregulated proteins in the sEVs_Tu_ were mainly enriched for the metabolic process and O‐glycan biosynthesis pathway (Figure 2D and Figure S2B, Supporting Information). We further screened glucose metabolism‐related proteins and NSP in the protein profiles through annotation from the database. As illustrated in the heatmap, GFAT1 is not only the protein in the glucose metabolism process with the greatest fold change (Figure 2E) but also a markedly upregulated NSP in sEVs_Tu_ (Figure 2F). These findings suggest that GFAT1 is enriched in BCa‐derived sEVs.

To further investigate the clinical significance of the secretory GFAT1 loaded in BCa‐derived sEVs (sEVs‐GFAT1), we detected the concentration of GFAT1 in urine‐derived sEVs (usEVs), that is, usEVs‐GFAT1, which were isolated from the urine samples of MIBC patients on the day before surgery (pre‐usEVs) and the 30th day after surgery (post‐usEVs) (Figure 2A–C). We found a prominent reduction in GFAT1 levels in the post‐usEVs (Figure 2G,H) and a marked positive correlation between the EC percentage in tumor tissues and the pre‐usEVs‐GFAT1 level (Figure S2C, Supporting Information). Conversely, post‐usEVs‐GFAT1 levels did not show a significant correlation (Figure S2D, Supporting Information). These collective results indicate that sEVs‐GFAT1 is a potential regulator in the TME, modulating the HBP‐related metabolism and angiogenetic function of ECs.

### 2.4 The Nutrient‐Deprived Intratumor Microenvironment Promotes GFAT1 Expression and sEV‐Mediated Secretion from Bladder Tumor Cells

**Figure 3:**
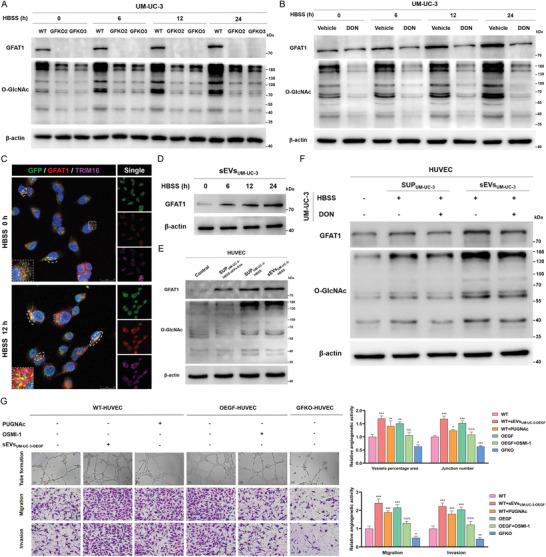
The nutrient‐deprived intratumor microenvironment enhances the secretion of sEVs‐GFAT1 from bladder tumor cells and induces angiogenesis. A) IB assays detected the expression of GFAT1 and O‐GlcNAc in UM‐UC3‐WT cells and UM‐UC‐3‐GFKO cells after time‐incremental HBSS incubation (6 to 24 h). B) IB results showing the expression of GFAT1 and O‐GlcNAc in UM‐UC‐3 cells treated with vehicle and DON following time‐increasing HBSS administration (6 to 24 h). C) IF assays recorded the expression, distribution, and colocalization of GFAT1 (red), TSG101‐GFP (green), and TRIM16 (purple) in UM‐UC‐3 after starvation treatment (12 h) or not. The white ellipses represent the secretory sEVs packaging GFAT1 (sEVs‐GFAT1, the colocalization of GFAT1, TSG101‐GFP, and TRIM16) accumulated at the cellular edge region. Scale bar: 25 µm. D) IB assays examined the GFAT1 levels in sEV‐derived UM‐UC‐3 cells after time‐incremental HBSS incubation (6 to 24 h). E) IB results showing the levels of GFAT1 and O‐GlcNAc in the HUVECs following administration of the sEV‐free supernatant of HBSS‐treated UM‐UC‐3 cells, the supernatant of HBSS‐treated UM‐UC‐3 cells, and sEVs derived from the supernatant of HBSS‐treated UM‐UC‐3 cells (30 µg sEVs per 2 × 10^6^ HUVECs; starvation length: 12 h). F) IB assays detected the levels of GFAT1 and O‐GlcNAc in the HUVECs after treatments with the supernatant and supernatant‐isolated sEVs of the UM‐UC‐3 cells treated with DON or not (starvation length: 12 h). G) Assessments of the abilities of tube formation, migration, and invasion of WT‐HUVECs, OEGF‐HUVECs, and GFKO‐HUVECs, after respective treatment with 50 µm PUGNAc, 50 µm OSMI‐1, and (or) sEVs derived from UM‐UC‐3‐OEGF (sEVs_UM‐UC‐3‐OEGF_) for 24 h. Scale bar: 80 µm. The result was normalized according to the result of WT. *** *p* < 0.001, ** *p* < 0.01, and * *p* < 0.05 represent significant differences compared with WT; ^%%%^ *p* < 0.001 and ^%%^ *p* < 0.01 represent significant differences compared with OEGF; one‐way ANOVA followed by Tukey's test.

To further elucidate the secretory mechanism of sEVs‐GFAT1, we first established GFKO and OEGF stable cell lines and found that the variations in GFAT1 levels in BCa cells and sEVs were synergistic, implying that the expression of GFAT1 in tumor cells directly influences the sEVs‐GFAT1 level in the TME (Figure S3A–D, Supporting Information). Intriguingly, our previous study found that the nutrient‐scarce intratumor microenvironment, on the one hand, causes aberrant protein profiles in BCa cells and, on the other hand, facilitates secretory autophagy‐induced sEV secretion.^[^ ^31^ ^]^ Therefore, we then established a nutrient‐deprived model in vitro (Figure S4A–D, Supporting Information) and detected the gradually increased expression levels of GFAT1 and O‐GlcNAc in BCa cells as the starvation time increased, while the accumulation was impeded in GFKO cells (**Figure** **3**A) or cells treated with DON (Figure 3B). Additionally, we observed much more colocalization of GFAT1 and secretory sEVs identified by TSG101‐GFP^[^ ^46^ ^]^ and TRIM16^[^ ^47^ ^]^ in the BCa starvation model than in fed cells, and these secretory sEVs‐GFAT1 predominantly accumulated at the cellular edge (Figure 3C). Simultaneously, we detected that the increase in GFAT1 levels in the sEVs was also time dependent, consistent with the intracellular alteration (Figure 3D). These results suggest that the nutrient‐deprived intratumor microenvironment provokes GFAT1 expression and sEV‐mediated secretion from bladder tumor cells.

### 2.5 sEVs‐GFAT1 Promotes Angiogenesis by Increasing O‐GlcNAcylation in ECs

To gain further insights into the effect and mechanism of sEVs‐GFAT1 in ECs in the TME, we established a series of in vitro models to simulate the TME and validate whether sEVs‐GFAT1 heighten HBP‐mediated O‐GlcNAcylation in ECs and thus promote tumor angiogenesis (Figure S4E, Supporting Information). After treating HUVECs with BCa cell‐derived culture supernatant and sEVs, we found that the levels of GFAT1 and O‐GlcNAc in HUVECs were strikingly higher, while the increase was notably blunted when sEVs were eliminated from the supernatant (Figure 3E). In addition, incubation with culture supernatant and sEVs derived from BCa cells in which GFAT1 expression was suppressed by DON caused a synergistic decrease in GFAT1 and O‐GlcNAc levels in HUVECs, consistent with the results obtained in BCa cells (Figure 3F). Therefore, we conclude that BCa‐secreted GFAT1 via sEVs evokes the increase in levels of GFAT1 and O‐GlcNAcylation in ECs.

Next, we explored whether and how sEVs‐GFAT1 modulates the angiogenetic activity of ECs. As illustrated in Figure 3G, we observed that tube formation, migration, and invasion were considerably potentiated when GFAT1 was overexpressed or when cells were administered UM‐UC‐3‐OEGF cell‐derived sEVs but sharply diminished in GFKO HUVECs. Then, we employed OGT and O‐GlcNAcase (OGA) inhibitors to validate whether O‐GlcNAcylation plays a dominant role in GFAT1‐mediated angiogenetic regulation. Exactly as expected, elevating the O‐GlcNAcylation level comprehensively augmented angiogenetic abilities; conversely, inhibition of O‐GlcNAcylation reversed the increasing angiogenesis caused by GFAT1 overexpression. These findings collectively imply that sEVs‐GFAT1 promote angiogenesis by strengthening O‐GlcNAcylation.

### 2.6 GFAT1‐Strengthened SerRS O‐GlcNAcylation at Ser101 Promotes Angiogenesis

**Figure 4:**
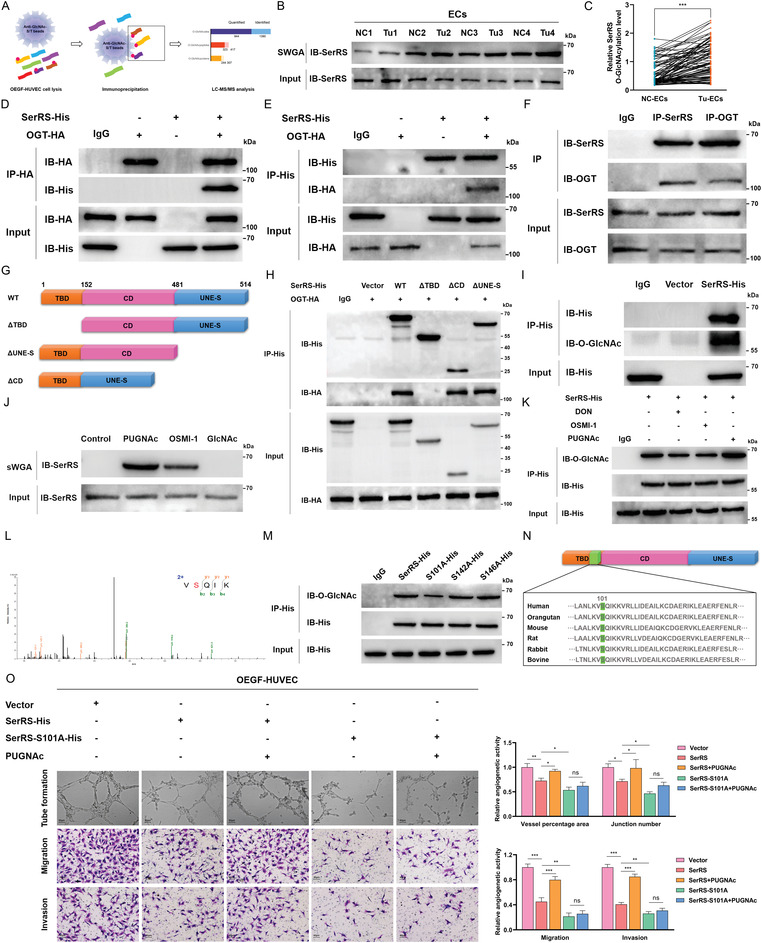
sEVs‐GFAT1 promotes angiogenesis by strengthening SerRS O‐GlcNAcylation at Ser101 in ECs. A) Flowchart delineating the processes and results of O‐GlcNAcylation LC‐MS/MS analysis. B) sWGA pull‐down assays were performed in paired NC‐ECs and Tu‐ECs of MIBC patients (*n* = 96). IB was determined using anti‐SerRS (left). C) The relative SerRS O‐GlcNAcylation levels were quantitatively analyzed by paired 2‐tailed Student's *t*‐test (right). ***p < 0.001. Co‐IP assays of SerRS‐His and OGT‐HA were detected using an anti‐HA antibody (D) or an anti‐His antibody (E) in 293T cells. F) Co‐IP assays of endogenous SerRS and OGT in HUVECs. G) Schematic representation of the SerRS construct. WT SerRS contains three domains, including a tRNA binding domain (TBD), a catalytic domain (CD), and a unique carboxyl‐terminal domain (UNE‐S). Truncation mutants of SerRS, comprising amino acids 152–514 (ΔTBD), 1–481 (ΔUNE‐S), or the full‐length removal of 152–481 (ΔCD). H) Co‐IP assays of interactions between OGT and WT SerRS, the ΔTBD, the ΔCD, or the ΔUNE‐S in 293T cells. I) SerRS IP assays with anti‐His antibody in 293T cells transfected with SerRS‐His or a vector control. IB was determined using anti‐O‐GlcNAc and anti‐His. J) sWGA pull‐down assays were performed in HUVECs treated with 50 µm PUGNAc or 50 µm OSMI‐1 for 24 h. IB was detected by anti‐SerRS. K) Cell lysates of the HUVECs treated with 50 µm PUGNAc, 50 µm OSMI‐1, or 20 µm DON were immunoprecipitated with anti‐His antibody and immunoblotted. L) MS analysis identified residue Ser101 as the SerRS O‐GlcNAcylation site. M) IP assays with anti‐His antibody in the HUVECs transfected with vectors, respectively, containing full‐length SerRS‐His, SerRS‐S101A‐His, SerRS‐S142A‐His, or SerRS‐S146A‐His. N) Sequence alignment of SerRS nearby Ser101 among multi‐species. O) The capacities of tube formation, migration, and invasion of OEGF‐HUVECs transfected with a vector control, full‐length SerRS‐His, or SerRS‐S101A‐His after administration of 50 µm PUGNAc or not. Scale bar: 80 µm. The result was normalized according to the result of Vector. **p < 0.01; *p < 0.05; ns represents no significant difference; one‐way ANOVA followed by Tukey's test.

To further investigate how GFAT1‐augmented protein O‐GlcNAcylation promotes angiogenesis, we performed IP coupled with tandem LC–MS/MS analysis to identify O‐GlcNAcylated proteins specifically interacting with GlcNAc‐S/T in OEGF‐HUVECs (**Figure** **4**A). Finally, 944 O‐GlcNAcylated sites, 323 O‐GlcNAcylated peptides, and 244 O‐GlcNAcylated proteins were identified and quantified, of which SerRS was strikingly enriched for the angiogenetic pathway (Figure 4A). To validate the clinical significance of SerRS O‐GlcNAcylation in ECs, we compared the levels of O‐GlcNAcylated SerRS in NC‐ECs and Tu‐ECs and detected increases in Tu‐ECs to varying degrees, analogous to the alteration of GFAT1 levels (Figure 4B,C). Next, we verified the O‐GlcNAc modification of SerRS in vitro. As shown in Figure 4D–F, interactions between SerRS and OGT were confirmed by co‐IP assays in 293T cells and HUVECs. To detect the precise domain(s) of SerRS required for interacting with OGT, we established and transfected the full‐length HA‐tagged OGT plasmid and different His‐tagged fragments of SerRS plasmids into 293T cells (Figure 4G). We found that OGT no longer interacted with SerRS devoid of the N‐terminal tRNA binding domain (TBD), whereas SerRS lacking catalytic domain (CD) or unique carboxyl‐terminal domain (UNE‐S) illustrated a strong interaction with OGT (Figure 4H).

Next, we ascertained whether SerRS was modified by O‐GlcNAc in HUVECs and 293T cells. As shown in Figure 4I, we detected an obvious interaction between SerRS and O‐GlcNAc in 293T cells. Consistently, the results of SWGA pull‐down assays also demonstrated that endogenous SerRS could be modified by O‐GlcNAc in HUVECs, and the modification was facilitated by PUGNAc (an OGA inhibitor) and attenuated when the O‐GlcNAcylation process was inhibited by OSMI‐1 (an OGT inhibitor) or GlcNAc (Figure 4J). Additionally, we found that administration of OSMI‐1 or the inhibition of GFAT1 expression by DON also caused a decrease in levels of exogenous SerRS O‐GlcNAcylation in HUVECs, while PUGNAc prominently increased levels of this modification (Figure 4K).

Then, we validated the O‐GlcNAcylated sites identified by MS analysis of SerRS. As illustrated in Figure 4L, serine 101 (Ser101) was the primary O‐GlcNAcylated site of SerRS. Next, we established site‐specific mutants of SerRS using alanine to replace three potential O‐GlcNAcylated sites (S101A, S142A, and S146A). The results showed that levels of SerRS O‐GlcNAcylation were sharply reduced when Ser101 was replaced, implying that Ser101 is the main site on SerRS responsible for the O‐GlcNAc interaction (Figure 4M). Importantly, we observed high conservation of the residue Ser101 and its surrounding amino acids among vertebrates, denoting the evolutionarily conserved effect of Ser101 in the regulation of SerRS (Figure 4N).

Finally, we detected the role of SerRS O‐GlcNAcylation at Ser101 in angiogenesis. As shown in Figure 4O, exogenous parental SerRS considerably attenuated the angiogenic activities of OEGF‐HUVECs, including tube formation, migration, and invasion, whereas the suppressive role was dramatically reversed when the O‐GlcNAcylation level was increased by PUGNAc. More importantly, the provoking effect of PUGNAc is sharply weakened when Ser101 is mutated to alanine. Additionally, in OEGF‐HUVECs without PUGNAc treatment, the exogenous S101A mutation led to even lower angiogenic capabilities than those observed with the parental SerRS. Taken together, these findings suggest that GFAT1‐mediated elevation of SerRS O‐GlcNAcylation levels at Ser101 improves the angiogenic activities of ECs.

### 2.7 SerRS O‐GlcNAcylation at Ser101 Decreases Its Stability by Increasing Ubiquitination

**Figure 5:**
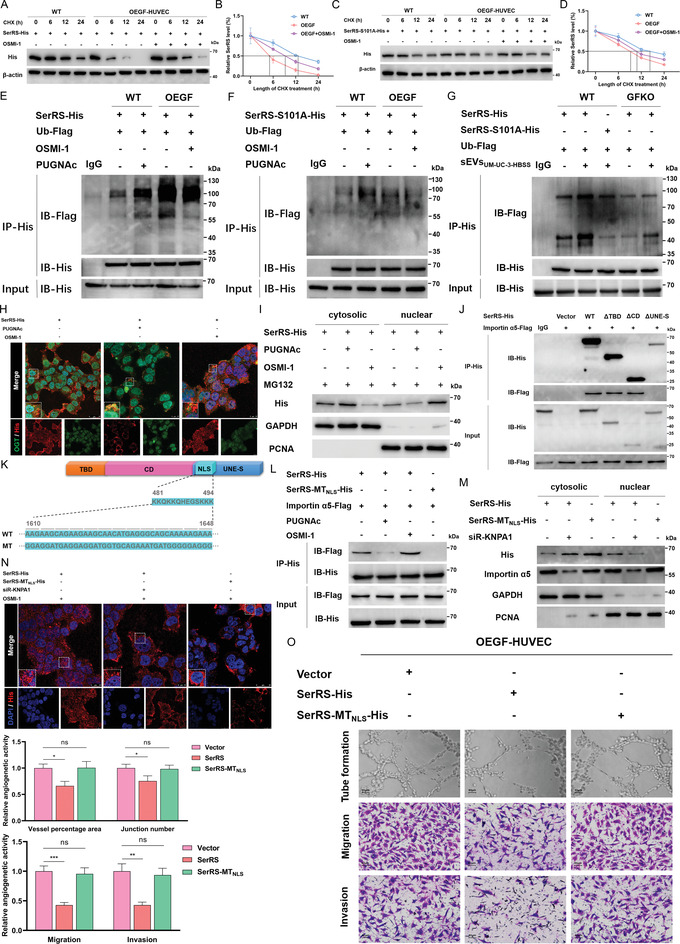
SerRS O‐GlcNAcylation at Ser101 decreases its stability by inducing ubiquitination and impedes importin *α*5‐mediated nuclear translocation. Half‐life detection and quantitative analysis of the SerRS‐His (A,B) and the SerRS‐S101 mutant (C,D) in parental HUVECs and OEGF‐HUVECs following treatments with 50 µm PUGNAc or 50 µm OSMI‐1. The parental SerRS (E) and the SerRS‐S101A mutant (F) ubiquitination in parental HUVECs and OEGF‐HUVECs in the presence of Flag‐tagged ubiquitin (Ub‐Flag). G) The parental SerRS and the SerRS‐S101A mutant ubiquitination in parental HUVECs and GFKO‐HUVECs transfected with Ub‐Flag after administration of sEVs derived from HBSS‐treated UM‐UC‐3 cells (starvation length: 12 h). H) IF results illustrating the expression, subcellular localization, and colocalization of SerRS‐His (red) and OGT (green) in HUVECs transfected with SerRS‐His following treatments with vehicle, 50 µm PUGNAc or 50 µm OSMI‐1. Scale bar: 25 µm and 10 µm. I) IB showing the levels of His‐tagged SerRS in the cytosolic and nuclear of HUVECs treated with 40 µm MG132 and 50 µm PUGNAc either or 50 µm OSMI‐1. J) Co‐IP assays of interactions between importin *α*5‐Flag and WT SerRS, the ΔTBD, the ΔCD, or the ΔUNE‐S in 293T cells. K) Schematic representation of the wild‐type NLS and the NLS mutant in SerRS. L) IP assays with anti‐His antibody in the HUVECs transfected with a vector importin *α*5‐Flag following treatments with vehicle, 50 µm PUGNAc, or 50 µm OSMI‐1. M) IB assays detected the levels of the His‐tagged SerRS or the SerRS‐MT_NLS_ mutant in the cytosolic and nuclear of HUVECs treated with siRNA‐KPNA1 or not. N) IF results showing the expression and subcellular localization of the His‐tagged SerRS or the SerRS‐MT_NLS_ mutant (red) in HUVECs in the presence of 50 µm OSMI‐1 and (or) siRNA‐KPNA1. Scale bar: 25 and 10 µm. O) The abilities of tube formation, migration, and invasion of OEGF‐HUVECs transfected with a vector control, full‐length SerRS‐His, or SerRS‐MT_NLS_‐His. Scale bar: 80 µm. The result was normalized according to the result of Vector. *p < 0.05; ns represents no significant difference; one‐way ANOVA followed by Tukey's test.

Next, we sought to explore the deeper mechanism by which SerRS O‐GlcNAcylation at Ser101 facilitates angiogenesis. Emerging studies have delineated the effect of O‐GlcNAcylation on protein stability.^[^ ^48^, ^49^ ^]^ We therefore performed a cascade of cycloheximide‐chase assays to examine the half‐life of SerRS. As shown in **Figure** **5**A,B, the half‐life of parental SerRS in the OEGF‐HUVEC was markedly shorter than that of WT, which could be partially reversed by OSMI‐1. In addition, induction of O‐GlcNAcylation with PUGNAc notably shortened the half‐life of SerRS in WT. Nevertheless, the reduction and extension of the half‐life caused by PUGNAc and OSMI‐1, respectively, became blunt when Ser101 was mutated to alanine (Figure 5C,D). Consistently, the increased GFAT1 levels in the HUVECs transfected with the S101A mutation did not contribute to the sharp curtailment of half‐life as the parental SerRS (Figure 5C,D). These results illuminate that O‐GlcNAcylation at Ser101 decreases the stability of SerRS. To gain mechanistic insights into the regulation of SerRS stability, we further explored whether O‐GlcNAcylation promoted proteasome‐mediated SerRS degradation. As expected, the level of interaction between parental SerRS and ubiquitin (Ub) in OEGF‐HUVECs was much higher than that in WT cells; more importantly, PUGNAc promoted the interaction, whereas OSMI‐1 had the opposite effect (Figure 5E). Furthermore, the IP results in Figure 5F show that the S101A mutation not only considerably diminishes the interaction with Ub but also mitigates the regulatory effects of PUGNAc and OSMI‐1. To further ascertain the effect of starving BCa cell‐derived sEVs on the O‐GlcNAcylation and ubiquitination of SerRS in HUVECs, we supplemented cells with sEVs_UM‐UC‐3‐HBSS_ and found that the interaction between parental SerRS and Ub was prominently augmented in both WT and GFKO cells, while the promoting effect on the S101A mutation was less obvious (Figure 5G). Together with these findings, we propose a convincing mechanism by which exogenous GFAT1 from BCa cell‐secreted sEVs increases the levels of O‐GlcNAc modification of SerRS at Ser101 and then promotes ubiquitination‐mediated degradation of SerRS in ECs.

### 2.8 SerRS O‐GlcNAcylation at Ser101 Impedes Its Nuclear Translocation Mediated by the Interaction of Importin *α*5 with the NLS Motif

It has been documented that the angiogenetic role of SerRS relies on its nuclear localization, which attenuates VEGFA expression.^[^ ^50^, ^51^ ^]^ Consequently, we detected whether SerRS O‐GlcNAcylation affects its nuclear translocation using an IF assay. Intriguingly, we found that administration of PUGNAc strikingly reduced the total levels of SerRS, especially in the nucleus, but we observed the opposite effect in HUVECs whose O‐GlcNAcylation was inhibited by OSMI‐1 (Figure 5H). More importantly, O‐GlcNAcylated SerRS was predominantly found outside the nucleus (Figure 5H), suggesting that O‐GlcNAcylation may affect the subcellular localization of SerRS. Therefore, we sought to probe the mechanistic relationship between the O‐GlcNAcylation and nuclear translocation of SerRS. The results of IB assays showed that PUGNAc notably reduced the SerRS level in the nucleus, accordingly leading to its accumulation in the cytoplasm. In contrast, we detected a considerably higher level of nuclear SerRS after OSMI‐1 treatment (Figure 5I).

Then, we focused on exploring how the O‐GlcNAc modification decreases the nuclear SerRS level. We inadvertently found the interaction of SerRS with importin *α*5, which functions in nuclear protein import as an adapter protein for nuclear receptor importin *β* (Figure 5J), suggesting that the nuclear translocation of SerRS may occur in an importin *α*5‐dependent manner. In addition, we detected that the interaction only occurred in the specific UNE‐S domain harboring a nuclear localization signal (NLS) (Figure 5J). To further validate importin *α*5‐mediated SerRS nuclear translocation and the binding specificity of importin *α*5 on the NLS motif, we performed site‐directed mutagenesis of the NLS motif in SerRS (SerRS‐MT_NLS_) and found that importin *α*5 no longer interacted with SerRS‐MT_NLS_ (Figure 5K). Furthermore, induction of O‐GlcNAcylation led to prominently the reduced interaction of SerRS and importin *α*5, whereas OSMI‐1 reversed this effect (Figure 5L). More persuasively, silencing karyopherin subunit alpha 1 (KPNA1, the gene encoding importin *α*5) partially decreased the SerRS level in the nucleus, while removal of the NLS motif completely eliminated nuclear SerRS expression and simultaneously aggravated its accumulation in the cytoplasm (Figure 5M). Accordingly, the results of IF assays also showed that, unlike the abundant nuclear localization of parental SerRS, SerRS‐MT_NLS_ was predominantly distributed in the perinuclear region, and siR‐KPNA1 caused less nuclear distribution of parental SerRS (Figure 5N). Then, we detected the significance of the NLS motif in SerRS‐caused suppression of angiogenesis. We detected that exogenous parental SerRS led to the prominent reductions of angiogenic activities, including tube formation, migration, and invasion, whereas the inhibiting effect of SerRS is dramatically reversed when the NLS motif is mutated (Figure 5O). Taken together, these results provide compelling evidence that SerRS O‐GlcNAcylation at Ser101 impedes importin *α*5‐mediated SerRS nuclear translocation by attenuating the interaction between importin *α*5 and the NLS motif, and SerRS nuclear translocation is crucial for its suppressing effect on angiogenesis.

### 2.9 SerRS O‐GlcNAcylation at Ser101 Increases VEGFA Transcription by Inhibiting the Nuclear Translocation of SerRS

**Figure 6:**
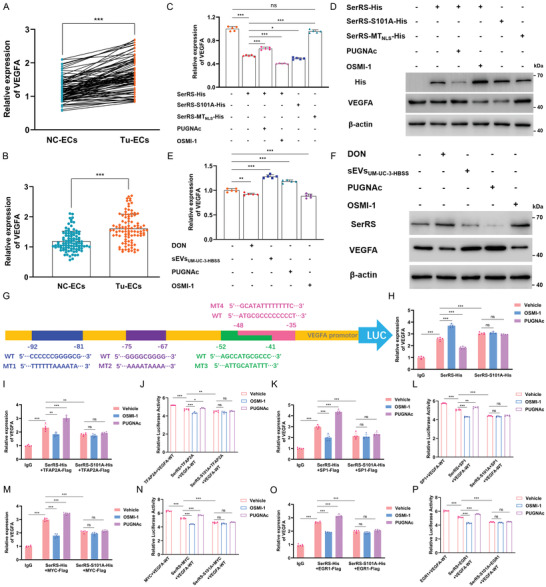
SerRS O‐GlcNAcylation at Ser101 impedes intranuclear SerRS‐induced VEGFA transcriptional repression. A,B) The expression of VEGFA at transcriptional level was compared between NC‐ECs and Tu‐ECs of MIBC patients (*n* = 96) by unpaired and paired 2‐tailed Student's *t*‐test. ***p < 0.001. The transcriptional (C) and translational (D) levels of VEGFA in HUVECs transfected with His‐tagged vectors, respectively, containing full‐length SerRS, the SerRS‐S101A mutant, and the SerRS‐MT_NLS_ mutant following administration of vehicle, 50 µm PUGNAC, or 50 µm OSMI‐1. *** *p* < 0.001, * *p* < 0.05; one‐way ANOVA followed by Tukey's test. The transcriptional (E) and translational (F) levels of endogenous VEGFA in HUVECs treated with sEVs_UM‐UM‐3‐HBSS_ (starvation length: 12 h), 20 µm DON, 50 µm PUGNAc, or 50 µm OSMI‐1. *** *p* < 0.001, ** *p* <0.01; one‐way ANOVA followed by Tukey's test. G) Schematic representation of the binding sites of TFs and SerRS on the GC‐rich region of the VEGFA proximal promoter. H) ChIP‐qPCR results showing the levels of VEGFA binding on the parental SerRS and the S101A mutant after administration of vehicle, 50 µm PUGNAC, or 50 µm OSMI‐1. ChIP‐qPCR results and luciferase reporter results illustrating the binding activities of TFAP2A (I,J), SP1 (K,L), MYC (M,N), and EGR1 (O,P) on the VEGFA proximal promoter in the presence of the parental SerRS‐His or the S101A‐His mutant, after administration of vehicle, 50 µm PUGNAC, or 50 µm OSMI‐1. ***p < 0.001, **p < 0.01, *p < 0.05; ns represents no significant difference.

These new insights into the stability and subcellular localization of O‐GlcNAcylated SerRS prompted us to explore whether and how the O‐GlcNAc modification guides SerRS‐mediated angiogenetic regulation. Given the indispensable role of VEGFA in vasculogenesis, we first compared the transcriptional and translational levels of VEGFA in Tu‐ECs and NC‐ECs. As shown in Figures 1J and **6**A,B, both the mRNA and protein levels of VEGFA in Tu‐ECs were notably higher than those in NC‐ECs, consistent with the difference in SerRS O‐GlcNAcylation (Figure 4B,C). Therefore, the focus turned to the possibility that SerRS O‐GlcNAcylation promotes VEGFA transcription. Interestingly, we found that exogenous supplementation with SerRS decreased the relative expression levels of VEGFA mRNA, and the reduction was further aggravated by OSMI‐1 but was strikingly weakened by PUGNAc (Figure 6C). Of special importance, the S101A mutation led to slightly higher SerRS‐evoked suppression than that achieved with the parental SerRS, whereas SerRS‐MT_NLS_ has almost no SerRS activity (Figure 6C). We also detected basically conformable results at the translational level (Figure 6D). Furthermore, we next investigated the effects of the O‐GlcNAc modification of endogenous SerRS on VEGFA expression. As illustrated in Figure 6E,F, attenuation of O‐GlcNAcylation using GFAT1 or OGT inhibitors resulted in a reduction in VEGFA levels, whereas supplementation with sEVs derived from starving BCa cells and administration of PUGNAc increased both the mRNA and protein levels of VEGFA to varying degrees. These findings collectively suggest that O‐GlcNAcylation at Ser101 impedes the NLS motif‐guided nuclear translocation of SerRS and consequently leads to the increase in VEGFA transcription levels.

### 2.10 Intranuclear SerRS Inhibits VEGFA Transcription by Competitively Binding to the GC‐Rich Region of the Proximal Promotor with Other GC‐TFs

On these premises, we next sought to investigate how intranuclear SerRS attenuates VEGFA transcription. Emerging evidence has revealed that the proximal promoter region from −109 to −38 bp is highly GC‐rich and is crucial for constitutive VEGFA promoter activity.^[^ ^52^, ^53^ ^]^ Nuclear SerRS was validated to transcriptionally inhibit VEGFA expression by binding to the VEGFA promoter region from −62 to −36 bp and recruiting NAD‐dependent protein deacetylase sirtuin‐2 (SIRT2) for epigenetic gene silencing.^[^ ^54^ ^]^ These results inspired us to raise an interesting possibility that nuclear SerRS binds to the proximal promoter of VEGFA, competing with those transcription factors (TFs) with binding specificities to this GC‐rich region (GC‐TFs), and then decreasing transcriptional activity.

To this end, we screened four potential TFs containing the sequences required for binding to the proximal promoter of VEGFA in the JASPAR database (Figure S5A, Supporting Information) and bidirectionally verified their binding specificities by a series of dual‐luciferase reporter assays and chromatin immunoprecipitation (ChIP) analyses (Figure 6G). The results suggested that TFAP2A (Figure S5B,C, Supporting Information), SP1 (Figure S5D,E, Supporting Information), MYC (Figure S5F,G, Supporting Information), and EGR1 (Figure S5H,I, Supporting Information) can, respectively, bind to the “CCCCCCGGGGCG,” “GGGGCGGGG,” “AGCCATGCGCCC,” and “ATGCGCCCCCCCCT” sites on the VEGFA proximal promoter and then promote its transcription. In addition, the results of the ChIP assay confirmed the binding specificity of SerRS on the VEGFA proximal promoter from −62 to −36 bp (Figure S5J, Supporting Information). More importantly, the number of SerRS molecules binding to the proximal promoter was significantly increased when the O‐GlcNAc modification was attenuated by OSMI‐1 but dropped after induction by PUGNAc. Consistently, the S101A mutation also caused a prominently increasing binding number (Figure 6H). These findings suggest that O‐GlcNAcylation reduces the number of intranuclear SerRS molecules that can specifically bind to the GC‐rich region of the VEGFA proximal promoter.

Next, we explored whether O‐GlcNAcylation‐regulated nuclear translocation of SerRS affects the binding activities of the GC‐TFs on the VEGFA proximal promoter. As expected, supplementation with exogenous parental SerRS, to varying extents, attenuated the binding activities of these GC‐TFs (TFAP2A, SP1, MYC, and EGR1), which was partially reversed by PUGNAc, while OSMI‐1 further aggravated the suppressive effect (Figure 6I–P). In addition, the inhibitory role of the S101A mutation in GC‐TF binding was more dramatic than that of the parental SerRS (Figure 6I–P). Together with the above results, we propose a new mechanism by which the O‐GlcNAc modification decreases the level of intranuclear SerRS, which inhibits VEGFA transcription by competitively binding to the GC‐rich region of the proximal promotor with other GC‐TFs, such as TFAP2A, SP1, MYC, and EGR1.

### 2.11 BCa‐Derived sEVs‐GFAT1 Facilitates HBP‐Mediated SerRS O‐GlcNAcylation in ECs and Promote Tumor Angiogenesis In Vivo

**Figure 7:**
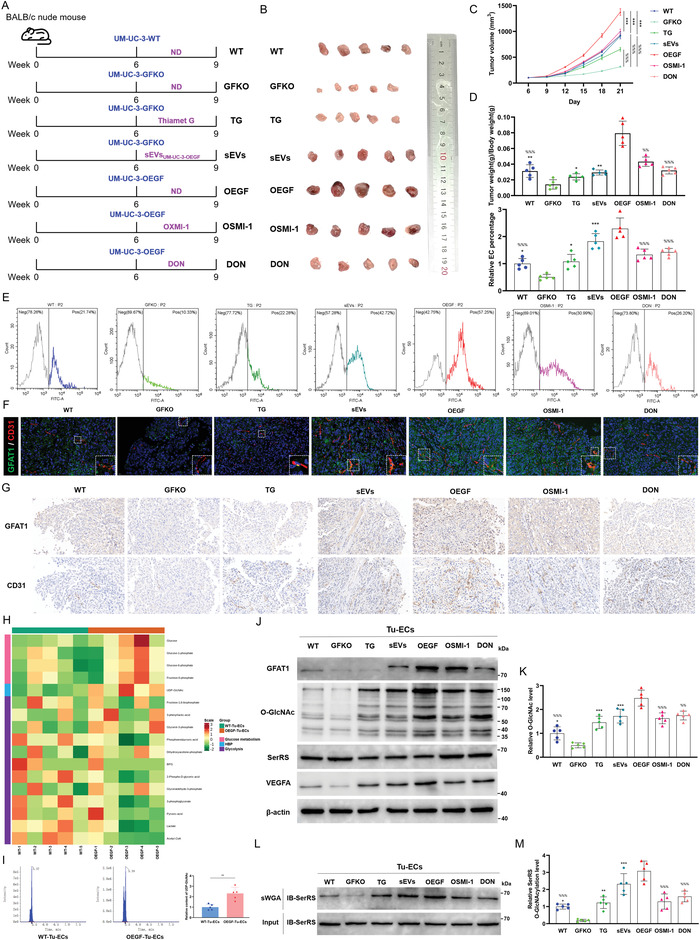
sEVs‐GFAT1 facilitates tumor angiogenesis in CDX nude mouse models of BCa. A) Schematic representation of the experimental protocols in vivo. UM‐UC‐3‐WT, UM‐UC‐3‐GFKO, and UM‐UC‐3‐OEGF were subcutaneously injected into the right thigh root of 6‐week‐old female BALB/c nude mice to establish CDX mouse models for BCa, as indicated, followed by administration of sEVs_UM‐UC‐3‐OEGF_ (50 µg/3 days), OSMI‐1 (1 mg/kg/2 days), thiamet G (20 mg kg^−1^ day^−1^), or DON (1 mg kg^−1^ day^−1^) for 3 weeks. ND represents mice were offered a normal diet. B) Gross appearances of neoplasia 21 days following subcutaneous injection (*n* = 5/group). C) Tumor volumes were measured at six time points. ***p < 0.001 represents a significant difference compared with GFKO; ^%%%^ *p* < 0.001 represents a significant difference compared with OEGF; one‐way ANOVA followed by Tukey's test. D) Tumor weight and body weight were measured at the end point. **p < 0.01 and *p < 0.05 represent significant differences compared with GFKO; ^%%%^ *p* < 0.001 and ^%%^ *p* < 0.01 represent significant differences compared with OEGF. E) The EC percentage in tumor tissues was detected by flow cytometry (down) and quantitatively analyzed by one‐way ANOVA followed by Tukey's test (top); the result was normalized according to the EC percentage of WT; ***p < 0.001 and *p < 0.05 represent significant differences compared with GFKO; ^%%%^ *p* < 0.001 represents a significant difference compared with OEGF. F) IF assays determining the expression of GFAT1 (green), and the number of vessel (marked by CD31, red) in tumor tissue slices. Scale bar: 20 µm. G) The indicated proteins in bladder tumors were evaluated by IHC assays of serial sections (scale bar: 20 µm). H) Glucose‐metabolite profiles, derived from Tu‐ECs of WT and OEGF mice, were detected using LC‐MS/MS metabolomics assays (*n* = 5/group). I) The relative contents of UDP‐GlcNAc in Tu‐ECs of WT and OEGF mice (*n* = 5/group) (*l*eft), and quantitative analysis (right). **p < 0.01. J) The indicated proteins in Tu‐ECs were assessed by IB assays. K) The O‐GlcNAc level was normalized according to the level of WT; ***p < 0.001 and *p < 0.05 represent significant differences compared with GFKO; ^%%%^ *p* < 0.001 and ^%%^ *p* < 0.01 represent significant differences compared with OEGF; one‐way ANOVA followed by Tukey's test. L) sWGA pull‐down assay was performed in Tu‐ECs of each group. IB was determined using anti‐SerRS. M) The level of SerRS O‐GlcNAcylation was normalized according to the level of WT; ***p < 0.001, **p < 0.01, and *p < 0.05 represent significant differences compared with GFKO; ^%%%^ *p* < 0.001 represents a significant difference compared with OEGF; one‐way ANOVA followed by Tukey's test.

Next, we established cell line‐derived xenograft (CDX) nude mouse models of BCa to further validate the above results in vivo (**Figure** **7**A). We found that knockout of GFAT1 considerably attenuated tumor growth, whereas intraperitoneal administration of TG and intravenous injection of sEVs derived from UM‐UC‐3‐OEGF cells both mitigated the suppression to varying degrees (Figure 7B–D). Reciprocally, high expression levels of GFAT1 evoke accelerated tumor growth, which can be inhibited following treatment with OSMI‐1 and DON (Figure 7B–D). In addition, we detected the EC percentage in the tumor by flow cytometry. In agreement with the trend of tumor growth, we examined the highest EC percentage in the tumors of OEGF mice, while OSMI‐1 mice and DON mice exhibited relatively lower EC percentages (Figure 7E). Consistently, administration of TG and sEVs_UM‐UC‐3‐OEGF_ partially reversed the downregulation of the EC percentage caused by GFAT1 knockout (Figure 7E). Furthermore, IF assays (Figure 7F) and IHC assays of serial sections (Figure 7G) showed analogous results. These results suggest that the elevated GFAT1 level confers an advantage for tumor angiogenesis in vivo.

Then, we isolated and purified the ECs from tumor tissues for further functional and mechanistic validations. The MS data show that the UDP‐GlcNAc level is considerably higher in the Tu‐ECs of OEGF mice than in WT mice, with the highest fold change, implying that the high expression level of GFAT1 in the tumor cells prominently promotes the HBP flux in the ECs (Figure 7H,I and Figure S6A, Supporting Information). Interestingly, the contents of some glucose metabolism‐related metabolites before the HBP branch (Figure S6B, Supporting Information) and in the glycolysis pathway (Figure S6C, Supporting Information) are not prominently different between WT and OEGF. Consistent with the in vitro and clinical data, the MS data in vivo suggest that GFAT1 triggers the glucose metabolic reprogramming by specifically accelerating the HBP flux in Tu‐ECs (Figure S6D, Supporting Information). Additionally, through IB assays, we found that the levels of GFAT1 in Tu‐ECs were altered in a generally parallel trajectory to those in tumor cells, and supplementation with sEVs_UM‐UC‐3‐OEGF_ evoked an increase in Tu‐ECs, while DON prominently reduced GFAT1 expression in Tu‐ECs (Figure 7J). Then, we detected almost synergistic alterations in the levels of O‐GlcNAc with those of GFAT1 in Tu‐ECs, which were further elevated following administration of TG and sEVs_UM‐UC‐3‐OEGF_ (Figure 7J,K). These in vivo results indicate that BCa‐derived sEVs‐GFAT1 in the TME increases the levels of GFAT1 and O‐GlcNAcylation in ECs. Accordingly, we found that the level of O‐GlcNAcylated SerRS was notably higher in the Tu‐ECs of OEGF mice and mice treated with sEVs_UM‐UC‐3‐OEGF_, whereas the Tu‐ECs of GFKO mice exhibited the lowest O‐GlcNAcylated SerRS level (Figure 7L,M). Consistent with the in vitro results of SerRS O‐GlcNAcylation, we detected that hyper‐O‐GlcNAcylation facilitates SerRS degradation and impedes SerRS‐induced VEGFA transcriptional repression, as evidenced by the expression of SerRS and VEGFA in Tu‐ECs (Figure 7J). In summary, these in vivo findings suggest that BCa‐derived sEVs‐GFAT1 reprogram glucose metabolism by increasing the HBP flux in ECs and promote angiogenesis by increasing levels of HBP‐mediated SerRS O‐GlcNAcylation.

**Figure 8:**
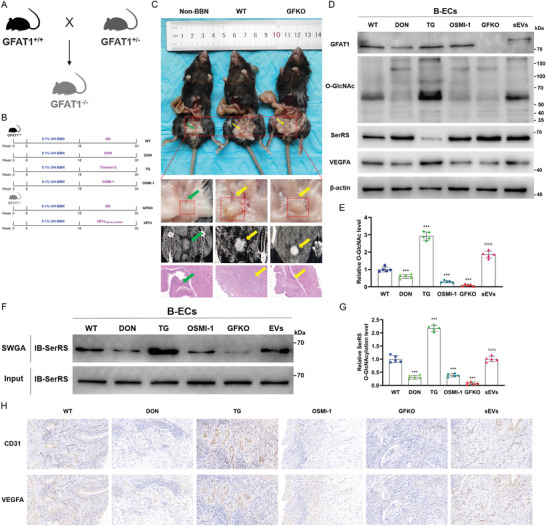
SerRS O‐GlcNAcylation in ECs promotes angiogenesis of orthotopic bladder tumors in GFAT1‐knockout mice. A) Reproductive strategy for generating GFAT1^–/–^ (GFKO) mice. B) Schematic representation of the experimental procedures in OH‐BBN‐induced orthotopic BCa models in GFAT1^–/–^ mice and wild‐type (GFAT1^+/+^) mice. C) Corresponding necropsy images (top and upper‐middle), computed tomography scans (lower‐middle), and HE staining (bottom) were collected at 30 weeks. Scale bar: 50 µm. Green and yellow arrows, respectively, indicate the normal bladder of untreated mouse and bladder tumors of OH‐BBN‐treated mouse. D) The indicated proteins in B‐ECs were assessed by IB assays. E) The O‐GlcNAc level was normalized according to the level of WT; ***p < 0.001 represents a significant difference compared with WT; ^%%%^ *p* < 0.001 represent a significant difference compared with GFKO; one‐way ANOVA followed by Tukey's test. F) sWGA pull‐down assay was performed in B‐ECs of each group. IB was determined using anti‐SerRS. G) The level of SerRS O‐GlcNAcylation was normalized according to the level of WT; ***p < 0.001 represents a significant difference compared with WT; ^%%%^ *p* < 0.001 represent a significant difference compared with GFKO; one‐way ANOVA followed by Tukey's test. H) The indicated proteins in bladder tumors were evaluated by IHC assays of serial sections. Scale bar: 20 µm.

In addition, we used OH‐BBN‐induced orthotopic BCa models in GFAT1^–/–^ mice and WT (GFAT1^+/+^) mice to further validate the role of BCa‐secreted GFAT1 via sEVs in the functional and mechanistic regulation of ECs (**Figure** **8**A,B). Consistent with the data from orthotopic BCa models in previous reports,^[^ ^55^ ^]^ results from serial computed tomography imaging with subsequent necropsy observation (Figure 8C) suggested that bladders in the mice treated with OH‐BBN had obvious tumor‐like lesions and larger volumes to varying degrees than the bladders of normal mice (Non‐BBN), indicating successful orthotopic neoplasia in the study. In addition, the results of HE staining (Figure 8C) showed that WT mice presented invasive bladder tumors but GFKO mice presented superficial bladder tumors, implying that GFAT1 promotes the invasion of bladder tumor.

Following the isolation and purification of ECs from mouse bladder tissues (B‐ECs), we detected that the expression levels of GFAT1 and O‐GlcNAc were strikingly attenuated in the B‐ECs of GFKO mice, leading to a relatively high level of SerRS and the dramatic suppression of VEGFA; however, sEVs_UM‐UC‐3‐OEGF_ weakened and even partially reversed the effects of GFAT1 knockout. Furthermore, inhibition of GFAT1 and O‐GlcNAcylation both elicited lower VEGFA levels and higher SerRS levels in B‐ECs than in WT mice, but TG had the opposite effect (Figure 8D,E). Accordingly, we examined that the alteration of the SerRS O‐GlcNAcylation level in the B‐ECs was basically consistent with the O‐GlcNAc change (Figure 8F,G). Importantly, through IHC assays (Figure 8H), we observed sparser vascular distribution (marked by CD31) and less expression of VEGFA in the tumors of GFKO mice than in WT mice, but sEVs_UM‐UC‐3‐OEGF_ partially reversed the effects of GFAT1 knockout. These findings in the orthotopic BCa models, consistent with the results of CDX models, provide compelling evidence that BCa‐secreted GFAT1 via sEVs increases the VEGFA level by increasing the O‐GlcNAc modification of SerRS in ECs, promoting tumor angiogenesis.

## 3 Discussion

**Figure 9:**
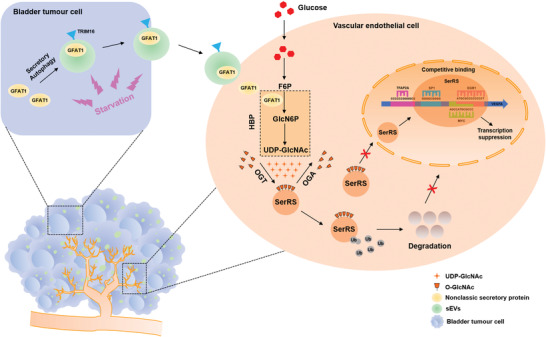
Proposed model for how BCa‐derived sEVs‐GFAT1 promote tumor angiogenesis. The starving intratumor microenvironment facilitates the secretion of BCa‐derived sEVs‐GFAT1, which forge a metabolic link between BCa cells and ECs, and improve angiogenetic activities by enhancing SerRS O‐GlcNAcylation in ECs.

Facing the fluctuating nutrient supply and interference due to insufficient and abnormal vasculature, tumor cells flexibly adapt their metabolic activities and establish metabolic symbiosis with various stromal cells in the TME through intercellular communications.^[^ ^56^, ^57^, ^58^ ^]^ Emerging evidence indicates that increased HBP flux and accordingly elevated protein O‐GlcNAcylation levels lead to endothelial dysfunction in the development of diabetic vasculopathies^[^ ^59^, ^60^ ^]^ and cardiovascular diseases.^[^ ^61^, ^62^ ^]^ Nevertheless, the mechanisms regulating tumor angiogenesis under metabolic stress conditions through HBP‐fuelled O‐GlcNAcylation are poorly understood. It remains unclear whether and how the TME remodels the HBP‐related metabolic process in ECs. Here, we present the first evidence that BCa‐derived GFAT1, through sEVs in the TME, prominently enhances HBP flux and increases O‐GlcNAcylation levels in ECs, which is pivotal to facilitating angiogenesis. Additionally, we discovered that SerRS O‐GlcNAcylation at Ser101 in ECs promotes its degradation by ubiquitination and impedes importin *α*5‐mediated nuclear translocation, thus reducing its competitive binding to the GC‐rich region of the VEGFA proximal promotor and mitigating transcriptional suppression (**Figure** **9**). Therefore, our research delineates a metabolic connection between BCa‐derived sEVs‐GFAT1 and the strengthened angiogenetic activity mediated by increased levels of O‐GlcNAcylation in ECs.

It is widely appreciated that tumor‐derived sEVs shuttling from cancer cells to stromal cells contribute to functional and mechanistic remodeling in the TME.^[^ ^63^, ^64^, ^65^ ^]^ Growing evidence indicates that sEVs deliver some key enzymes that regulate tumor neovascularization. For example, five‐fluorouracil‐resistant colon cancer cell‐derived sEVs containing dipeptidyl peptidase IV (DPP4) increased periostin expression levels in ECs and promoted angiogenesis by facilitating Twist1 nuclear translocation and inducing Smad pathway activation.^[^ ^66^ ^]^ Lysyl oxidase‐like 4 (LOXL4) is transferred to ECs by hepatocellular carcinoma cell‐derived sEVs, leading to angiogenesis by activating the FAK/Src signaling pathway.^[^ ^67^ ^]^ However, little is known about the roles of key metabolic enzymes delivered by tumor‐derived sEVs in the functional and mechanistic regulation of ECs. Here, for the first time, we identified that GFAT1 levels, the crucial rate‐limiting enzyme mediating the switch to the HBP branch, are increased in BCa‐secreted sEVs under nutrient‐deprived conditions and thereby reprograms glucose metabolism by increasing HBP flux in ECs. Interestingly, GFAT1 has been demonstrated to be barely detected in the ECs of healthy human tissues but exhibits increased levels in activated cells,^[^ ^68^, ^69^ ^]^ indicating that the aberrant expression and activity of GFAT1 in ECs may cause pathological dysfunction. Nevertheless, few studies have focused on the role of GFAT1 in angiogenetic modulation of ECs. In this study, we reveal a novel mechanism by which the accumulating exogenous GFAT1 from BCa‐derived sEVs drives strikingly increased HBP flux and O‐GlcNAcylation levels in ECs, leading to an increase in angiogenesis.

O‐GlcNAcylation is a dynamic, ubiquitous, and metabolism‐sensitive process that depends on two key enzymes (OGT and OGA) and the substrate UDP‐GlcNAc.^[^ ^70^ ^]^ Although the role of O‐GlcNAcylation in regulating protein homeostasis,^[^ ^48^, ^49^ ^]^ localization,^[^ ^71^, ^72^ ^]^ and transcriptional activity^[^ ^73^, ^74^ ^]^ has been widely appreciated, its role in the functional and mechanistic modulation of tumor angiogenesis remain unclear, especially under stress conditions, such as hypoxia and nutrient scarcity. The angiogenetic effect of O‐GlcNAcylation was mostly documented in diseases with vasculopathies, including idiopathic pulmonary arterial hypertension,^[^ ^75^ ^]^ retinopathy,^[^ ^76^ ^]^ and diabetes.^[^ ^77^ ^]^ To date, only a limited number of studies have characterized that increased O‐GlcNAcylation levels activate angiogenetic signaling pathways in tumor cells, and even fewer studies have focused on its role in the TME, such as in ECs. A recent study by Duan and colleagues documented that RACK1 O‐GlcNAcylation at Ser122 potentiated protein stability and ribosome binding and thereby promoted hepatocellular carcinogenesis and angiogenesis by increasing levels of EIF4E phosphorylation in hepatocellular carcinoma cells.^[^ ^78^ ^]^ Lynch et al. elucidated that increasing levels of O‐GlcNAcylation in prostate cancer cells caused increased invasion and angiogenesis by inducing the expression of matrix metalloproteinase (MMP)‐2, MMP‐9, and VEGF.^[^ ^79^ ^]^ However, in the present study, we demonstrate, for the first time, that the increasing HBP flux‐mediated increase in O‐GlcNAc modifications in ECs facilitates tumor angiogenesis.

Emerging evidence suggests a prominent role for SerRS in vascular development, independent of its well‐known role in aminoacylation regulation. For example, Shi and colleagues revealed that ATM/ATR‐mediated phosphorylation of SerRS at Ser101 and Ser241 decreased its DNA binding ability and thus attenuated hypoxia‐induced angiogenesis.^[^ ^43^ ^]^ Moreover, a work by Fu suggested the negative role of SerRS in angiogenesis by forming the SerRS/YY1 complex, which, while competing with NFKB1, binds distal cis‐regulatory elements of the VEGFA promoter.^[^ ^80^ ^]^ In this study, we present the first evidence that, in ECs, SerRS can be O‐GlcNAcylated at Ser101, accordingly leading to the suppression of its importin *α*5‐mediated nuclear translocation and its increased degradation by ubiquitination, which reduces its competitive binding to the VEGFA proximal promoter with other GC‐TFs and thus facilitates tumor angiogenesis. Although the role of SerRS in the transcriptional suppression of VEGFA has been previously illustrated,^[^ ^51^ ^]^ in the present research, we discovered the considerable competitive potential of SerRS for blocking the binding sites of the TFs possessing binding specificities on the GC‐rich region of the VEGFA proximal promoter.

It is well known that O‐GlcNAc modification is pivotal for protein subcellular localization and the accompanying functional regulation.^[^ ^71^, ^81^ ^]^ In line with our results, Carvalho and colleagues demonstrated that glucosamine‐induced O‐GlcNAcylation inactivated calnexin and decreased its level in the nucleus by inhibiting its phosphorylation.^[^ ^82^ ^]^ Zhang et al. also illuminated that ten‐eleven translocation protein 3 (TET3) O‐GlcNAcylation reduced its nuclear localization and thus attenuated its catalytic activity for 5‐hydroxymethylcytosine.^[^ ^83^ ^]^ However, a recent study by Tan proposed a different mechanism by which O‐GlcNAcylation of SRPK2 at the NLS motif promoted its nuclear translocation by inducing its interaction with importin *α*/*β*.^[^ ^84^ ^]^ We think the disparate O‐GlcNAcylated region may cause the difference in the role of importin *α*‐mediated nuclear translocation. Tan et al. detected that importin *α* preferred to bind to the O‐GlcNAcylated NLS region of SRPK2; however, Ser101, the primary O‐GlcNAcylated site of SerRS, is located away from the NLS motif.^[^ ^41^ ^]^ In addition, the work by Miura suggested that O‐GlcNAcylation did not regulate the nuclear translocation and phosphorylation of heat shock Factor 1.^[^ ^85^ ^]^ It is therefore plausible that O‐GlcNAc modulates protein nucleocytoplasmic shuttling in many ways, including through changing protein conformation,^[^ ^86^ ^]^ activating complex signaling pathways,^[^ ^87^ ^]^ and generating extensive crosstalk with other posttranscriptional modifications.^[^ ^88^, ^89^ ^]^ The need for a better understanding of the functions and mechanisms of O‐GlcNAcylation remains.

Angiogenesis is a fundamental process underlying BCa growth and progression. Although two drugs targeting angiogenesis (bevacizumab^[^ ^90^, ^91^ ^]^ and ramucirumab^[^ ^92^, ^93^ ^]^) for BCa have shown signs of antitumor activity in early‐stage clinical trials, the results have been instable, probably because of some other compensatory mechanisms.^[^ ^94^, ^95^ ^]^ Till now, no antiangiogenetic drugs are approved by Food and Drug Administration for BCa. Therefore, identifying more viable antiangiogenetic targets and corresponding patients with potentially good responses remain viable and necessary. In this study, we identify that GFAT1 drives an angiogenetic program in ECs, suggesting that targeting GFAT1 in GFAT1‐overexpressing patients may contribute to limit angiogenesis by decreasing the HBP flux and O‐GlcNAcylation in ECs.

In conclusion, we propose a novel metabolic link between BCa cells and ECs in the nutrient‐deprived TME, depending on BCa‐derived sEVs delivering key enzymes in the HBP. We demonstrate that the starving intratumor microenvironment facilitates the secretion of BCa‐derived sEVs containing GFAT1, which, as a pivotal metabolic switch, reprograms glucose metabolism by increasing the HBP flux in ECs and then enhances O‐GlcNAcylation. Additionally, the O‐GlcNAc modification of SerRS at Ser101 in ECs decreases its stability by increasing its ubiquitination and impedes its nuclear translocation mediated by importin *α*5, sequentially reducing the nuclear SerRS‐evoked transcriptional suppression of VEGFA and strengthening angiogenetic activities. Collectively, the present results provide a new perspective on the effect of BCa‐derived sEVs‐GFAT1 on metabolic reprogramming in the TME and expand our understanding of HBP‐induced SerRS O‐GlcNAcylation in ECs on promoting tumor angiogenesis, which may shed light on novel targets for BCa antiangiogenetic therapy.

## 4 Experimental Section

### Clinical Samples

A total of 220 treatment‐naïve BCa patients who underwent tumor excision or tissue biopsy between April 2019 and October 2021 in the First Affiliated Hospital of Chongqing Medical University were included in the study, of which 124 patients were T1N0M0 (nonmuscle‐invasive bladder cancer, NMIBC) and 96 patients were T2N0M0 (muscle‐invasive bladder cancer, MIBC). Patients with severe underlying diseases and other primary and metastatic cancers were excluded. Tumor tissues of all patients and normal urothelium tissues of MIBC patients were collected and used for EC isolation, and IHC, and IF analyses. Urine samples were collected from MIBC patients one day before surgery and 30 days after radical cystectomy to separate EVs. All participants provided written informed consent, and the study was approved by the Medical Ethics Committee of the First Affiliated Hospital of Chongqing Medical University (2021‐199). All clinical data were reviewed using medical records.

### Cell culture and treatment

The UM‐UC‐3 human urothelial carcinoma cell line and 293T cells were purchased from the Cell Bank of the Chinese Academy of Sciences (Shanghai, China). Human umbilical vein endothelial cells (HUVECs) were obtained from American Type Culture Collection (ATCC). HUVECs, UM‐UC‐3, and 293T cells were cultured in Dulbecco's modified Eagle's medium (DMEM, Gibco). All cell lines were supplemented with 10% fetal bovine serum (FBS, BioInd, Israel), 100 µg mL^−1^ streptomycin, and 100 U mL^−1^ penicillin at 37 °C in a humidified atmosphere of 5% CO_2_. The in vitro nutrient deprivation (starvation) model was established as described previously.^[^ ^96^, ^97^ ^]^ Cells were first washed with phosphate‐buffered saline (PBS, Solarbio, CHN) and then maintained in Hank's balanced salt solution (HBSS, Thermo Fisher Scientific, USA) for 6 to 24 h, as indicated in figures, in a humidified atmosphere of 5% CO_2_ at 37 °C. For in vitro sEV treatment, 2 × 10^6^ HUVECs were cultured in cell medium supplemented with 30 µg sEVs for 48 h (changed to 24 h during RNA detection). In addition, Thiamet G (Sigma–Aldrich, CHN), OSMI‐1 (Sigma–Aldrich, CHN), DON (MCE, CHN), or PUGNAc (Sigma–Aldrich, CHN) was added to the cell medium, as indicated.

### Lentivirus and CRISPR/Single Guide RNA Treatment

The lentivirus vector (pGLV5/Puro) containing the full‐length cDNA fragment of GFAT1 was transfected into UM‐UC‐3 cells and HUVECs, which were further screened with puromycin (5 µg mL^−1^) for 2 weeks to establish the stable overexpression cell line (OEGF). GFAT1‐knockout (GFKO) cells were established using the CRISPR–Cas9 system, as previously described.^[^ ^31^ ^]^ Individual guide sequences targeting GFAT1 were cloned into pSpCas9 BB‐2A‐Puro (PX459). The PX459 vector containing the GAL4‐glucose sequence was used as a control (mock). The sequences of oligonucleotides are listed in Table S1, Supporting Information.

### Mice

C57BL/6‐GFAT1^–/–^ mice were established by crossing C57BL/6 wild‐type (WT) mice and C57BL/6‐Gfat1^+/−^ mice (The Association for Assessment and Accreditation of Laboratory Animal Care‐approved SHANGHAI MODEL ORGANISMS, CHN). Four‐week‐old female BALB/c nude mice were obtained from AAALCA‐accredited SPF (Beijing) Biotechnology Co., Ltd. (Beijing, CHN). All mice were housed at room temperature (RT) with a 12‐h light/12‐h dark cycle at the standardized animal facility of the Animal Centre of Chongqing Medical University. All animal experiments complied with the Chongqing Medical University of Medicine Policy on the Care and Use of Laboratory Animals (199).

### Cell Line‐Derived Xenografts

Nude mice were randomized into six groups (*n* = 5/group). UM‐UC‐3 cells transfected with blank vector (WT) GFKO and OEGF cells were resuspended in cell medium (1 × 10^6^/100 µL) and subcutaneously injected into the right thigh root of 6‐week‐old female BALB/c nude mice. UM‐UC‐3‐OEGF‐derived sEVs resuspended in PBS were injected into the tail veins of mice in the sEVs group (50 µg/3 days). Mice in the OSMI‐1 group were administered OSMI‐1 (1 mg kg^−1^) by tail vein injection every other day. Mice in the TG group were intraperitoneally administered Thiamet G (20 mg kg^−1^) daily. Mice in the DON group were injected intraperitoneally with DON (1 mg kg^−1^) every other day. Body weight and tumor volume (V = 1/2 × length × width^2^) were monitored for 21 days after xenografts. After 3 weeks, peripheral blood was collected from each mouse via the right orbital vein and used for sEV isolation. Then, the mice were sacrificed, and the tumor tissues were separated for further histological examination and preparation of single‐cell suspensions.

### Orthotopic Mouse BCa Model

Because N‐butyl‐N‐4‐hydroxybutyl nitrosamine (OHBBN, TCI, JPN)‐induced bladder tumors exhibit heterogeneous histological features, including basal‐like, luminal‐like, and epithelial mesenchymal transition‐like cellular morphologies, OHBBN was used to establish an orthotopic BCa model, as previously described.^[^ ^55^, ^98^, ^99^ ^]^ C57BL/6‐GFAT1^–/–^ mice and C57BL/6 WT mice were randomized into two groups and four groups (*n* = 5/group), respectively. Each female mouse was supplied ad libitum with tap water containing 0.1% OHBBN for 12 weeks, starting from 6 weeks of age, and kept for an additional 12 weeks with regular water combined with different administrations among groups. Bottles were refreshed twice a week. Mice were inspected weekly for signs of distress associated with bladder lesions, such as hematuria. Mice in the sEVs group and OSMI‐1 group were intravenously injected via the tail vein with UM‐UC‐3‐derived sEVs (50 µg/3 days) and OSMI‐1 (1 mg/kg/2 days), respectively. Mice in the TG group were intraperitoneally administered Thiamet G (20 mg kg^−1^) daily. Mice in the DON group were injected intraperitoneally with DON (1 mg/kg/2 days).

Before harvest of the bladder in the 30th week, serial mCT scans were acquired 10 min following the administration of Gadopentetic acid (0.3 mmol kg^−1^, MCE, CHN). The images were captured by serial computed tomography scans. Then, the mice were sacrificed, and the bladders were removed and dissected sagittally into two parts, one of which was fixed in 4% paraformaldehyde for paraffin embedding, hematoxylin‐eosin (HE) staining, and IHC assays, while the other part was used to prepare a single‐cell suspension.

### Tissue and Single‐Cell Suspension Processing

Mouse tumor tissues and fresh human specimens were obtained from clear surgical fields where grossly apparent tumors were present or normal bladders were found to be unaffected by tumors, and the samples were dissected and transported at RT immersed in RPMI‐1640 medium with 10% FBS. Tissue samples were cut into approximately 1 mm^3^ pieces and enzymatically digested in HBSS buffer containing 1 mg mL^−1^ collagenase type 4, 1 mg mL^−1^ diapase type 2, and 30 U mL^−1^ DNase for 1 h on a rotor at 37 °C. Following filtration by a 70 mm Cell‐Strainer (BD, USA) in precooled HBSS buffer containing 0.5% bovine serum albumin (BSA, Sigma, USA), the cell suspension was centrifuged at 400 g and 4 °C for 5 min. The pelleted cells were resuspended in red blood cell lysis buffer (Solarbio, CHN) and incubated on ice for 5 min to lyse red blood cells followed by washing twice with precooled PBS. The cells were eventually resuspended in precooled HBSS buffer containing 0.5% BSA to make single‐cell suspensions.

### EC Isolation and Purification

To isolate and purify ECs, a single‐cell suspension was subjected to magnetic‐activated cell sorting using anti‐CD31 antibody‐conjugated magnetic beads according to the instructions of the CD31 MicroBead Kit (Miltenyi Biotech, GER). After immunolabeling with CD31 MicroBeads, the cells were loaded onto a column placed in the magnetic field of a MACS Separator. The magnetically labeled CD31^+^ cells were retained on the column, and the unlabeled cells ran through. After removal of the column from the magnetic field, the magnetically retained CD31^+^ cells (ECs) were eluted and analyzed for purity on a CytoFLEX (Beckman Coulter, USA).

### Flow Cytometry

To detect the percentage of ECs in tumor and normal urothelium tissues, 1 million cells of each sample were first blocked with Fc Receptor Blocking Solution (Biolegend, USA), followed by staining in precooled HBSS buffer containing 0.5% BSA and anti‐CD31 (Biolegend, USA) for 30 min at 4 °C and two washes. The stained cell suspensions were analyzed on a CytoFLEX.

### sEV Separation and Concentration from Cell Medium by Differential Ultracentrifugation

According to the guidelines of MISEV2018 of the Journal of Extracellular Vesicles,^[^ ^46^ ^]^ sEVs were depleted from complete medium containing basic medium, 10% FBS, and 1% penicillin–streptomycin by ultracentrifugation (UC) at 140 000× *g* for 18 h (Type 45 Ti rotor, k‐Factor 217.6, Beckman Coulter, USA). The cell line was cultured in EV‐free complete medium at 37 °C with 5% CO_2_. After 80–90% confluency, there were approximately 5 × 10^7^ cells per 15‐cm dish. Cells were processed with different treatments according to experimental requirements, and then the conditioned medium was harvested for sEV separation. There were approximately 5–10% dead cells at the time the conditioned medium was harvested.

sEV separation from the cell medium by differential UC was performed following a previously described method with minor modifications.^[^ ^100^ ^]^ Briefly, the cell medium was subjected to sequential centrifugation steps of 300 × *g* for 10 min, 2000 × *g* for 20 min, and 12 000 × *g* for 30 min to remove cells and cellular debris. The resulting supernatants were separately filtered by gravity through a 0.22 µm hydrophilic syringe filter (Millipore, MA, USA) to eliminate large cell debris. After 70 min of UC at 140 000 × *g* (Type 45 Ti rotor, adjusted k‐Factor 133, maximal acceleration, maximal deceleration), the pellet was gently resuspended in sterile PBS and was then ultracentrifuged again at 140 000 × *g* for 70 min to pellet the sEVs. Next, the sEV pellet was resuspended in 150 µL sterile PBS for subsequent analyses or stored at −80 °C until use. The number of freeze–thaw cycles was limited to a maximum of one.

### Urine‐Derived sEV Separation and Concentration by OptiPrep Density Gradient Ultracentrifugation

According to the guidelines of the ISEV,^[^ ^101^ ^]^ midstream urine specimens (100 mL) were collected from MIBC patients on the day before surgery and the 30th day after surgery. Before usEV separation, urine samples were preprocessed as follows: Urine samples were precleaned by centrifugation at 2000 × *g* for 20 min. The supernatant was collected and recentrifuged at 12000 × *g* and 4 °C for 30 min. The supernatant was collected again and filtered through a 0.22 µm hydrophilic syringe filter. The fresh urine samples following preprocessing (preprocessed urine samples) were directly used for usEV separation and stored at −80 °C.

Urine‐derived sEV separation and concentration were conducted by the OptiPrep density gradient ultracentrifugation method as previously described.^[^ ^102^ ^]^ Solutions of 5%, 10%, and 20% iodixanol were made by mixing appropriate amounts of homogenization buffer (0.25 m sucrose, 1 mm EDTA, 10 mm Tris‐HCl [pH 7.4]) and iodixanol working solution, which were prepared by combining the working solution buffer (0.25 m sucrose, 6 mm EDTA, 60 mm Tris‐HCl, [pH 7.4]) and the OptiPrepTM stock solution (60% w/v aqueous iodixanol solution, Axis‐Shield, Oslo, Norway). First, urine samples (50 mL) were concentrated to 500–800 µL using a 10 kDa centrifugal filter device (Centricon Plus‐70, Merck Millipore). Tris buffer (10 mm Tris‐HCl, pH: 7.4, 1 mm EDTA, and 0.25 M sucrose) was used to bring the volume of concentrated urine sample to 800 µL. Second, the 800 µL concentrated urine sample was resuspended in 3.2 mL working solution to prepare the 40% iodixanol suspension, which was layered on the bottom of a 17 mL Thinwall Polypropylene Tube (Beckman Coulter, USA). Next, the discontinuous bottom‐up OptiPrep density gradient (ODG) was prepared by overlaying the urine suspension with 4 mL 20% iodixanol, 4 mL 10% iodixanol, 3.5 mL 5% iodixanol, and 1 mL PBS in a 17 mL Thinwall Polypropylene Tube. The ODG system was centrifuged at 100 000 × *g* (acceleration: max; deceleration: 9) and 4 °C for 18 h (SW 32.1 Ti rotor with ravg = 11.36 cm and adjusted k‐factor = 298.0). Afterward, 1 mL of each ODG fraction was collected from top to bottom, and fractions 1–5, 6–10, and 11–16 were pooled into three tubes. Next, PBS was used to dilute the pooled fractions to reach 16 mL, and the solution was separately loaded in a new 17 mL Thinwall Polypropylene tube and centrifuged at 100 000 ×*g* (acceleration: max; deceleration: max) and 4 °C for 3 h (SW 32.1 Ti rotor with ravg = 11.36 cm and adjusted k‐factor = 298.0). The resulting pellets were resuspended in 100 µL PBS, and usEVs derived from the 6–10 fractions were mainly used for subsequent analysis or stored at −80 °C. Frozen preprocessed urine samples were thawed at room temperature and vortexed in advance.

### Transmission Electron Microscopy

Transmission electron microscopy (TEM, Hitachi‐7500, Yokohama, Japan) was used to observe sEV morphology. First, a 30 µL sEV sample was dropped onto a 100‐mesh copper grid and dried by filter paper after 10 min. Then, the grid was stained with phosphotungstic acid for 15 s and dried at room temperature.

### Nanoparticle Tracking Analysis

Nanoparticle tracking analysis was conducted to measure the size distribution of sEVs by ZetaView PMX 110 (Particle Metrix, Germany) and its bundled software (ZetaView 8.02.28). First, sEV samples were diluted in PBS to reach the recommended concentration for measurement (20–30 particles/frame). Then, 11 positions were measured throughout the particle, with at least two reading cycles at each position. After automatic analysis and removal of outlier positions, all data were further analyzed using the software. The software settings for analysis were as follows: the detection threshold was 3, the temperature ranged from 20 to 23 °C, the frame number was 30, and the measurement time was 30 s.

### Immunoblotting

Four categories of markers were detected in all bulk sEV preparations to demonstrate the presence of sEVs and assess their purity from common contaminants, as described in MISEV2018 guidelines^[^ ^46^ ^]^: i) Transmembrane or GPI‐anchored proteins associated with plasma membrane and/or endosomes: CD9; ii) cytosolic proteins recovered in EVs: TSG101; iii) major components of non‐EV coisolated structures: Tamm‐Horsfall protein uromodulin (UMOD); iv) transmembrane, lipid bound, and soluble proteins associated with intracellular compartments other than PM/endosomes: calnexin.

### Flow NanoAnalyzer

The absolute concentration and high‐resolution size distribution of sEVs was determined by Flow NanoAnalyzer in a NanoFCM system (NanoFCM, CHN).

### Tracking of sEVs

sEVs labeled with PKH67 (Sigma–Aldrich, CHN) were added into the culture medium of HUVECs for 12 h and 24 h. Images were obtained using laser confocal microscopy (Leica Microsystems AG).

### Plasmids and Transfection

Human cDNA of O‐GlcNAc transferase (OGT; UniProt ID: O15294), SerRS (UniProt ID: P49591), Ub (UniProt ID: P0CG47), and importin *α*5 (UniProt ID: P52294) was amplified by PCR using the human complementary DNA library. Full‐length cDNA, cDNA encoding certain residues, and mutant cDNA were subcloned into the pcDNA 3.1 vector (cloning sites: KpnI/BamHI or KpnI/XhoI) or pEGFP‐N1 vector (cloning sites: EcoRI/SacII) with different tags as indicated. Plasmids were verified by restriction digestion and DNA sequencing and were transfected using Lipofectamine 3000 (Invitrogen, USA) in serum‐free medium.

### Metabolite Detection and Analysis

To extract metabolites and remove proteins, ECs isolated from tissues were first washed with precooled PBS twice and then resuspended in a 500 µL mixture of the same volume of cold methanol and acetonitrile (Merck, GER). Next, the cell suspension was centrifuged at 14 000 × *g* and 4 °C for 20 min, and then 300 µL supernatant was transferred into a new centrifuge tube, which was incubated at −20 °C for 30 min. Then, the supernatant was recentrifuged at 14 000 × *g* and 4 °C for 10 min, and 200 µL of supernatant was transferred through a protein precipitation plate for further LC–MS analysis. Targeted metabolomics profiling of CD31^+^ cells and targeted metabolite detection were performed using an LC–ESI–MS/MS system (UPLC, ExionLC AD, https://sciex.com.cn/; MS, QTRAP 6500+ System, https://sciex.com/).

The concentrations of amino acids and glucose in BCa cells and tissues were, respectively, determined by a micro amino acid content assay kit (Solarbio, China) and a glucose content assay kit (Solarbio, China) according to the manufacturer's instructions.

### sEV Protein Identification by LC–MS/MS Analysis

sEVs were resuspended in lysis buffer (8 M urea, 1% protease inhibitor cocktail (Bimake, CHN)) and sonicated three times on ice using a high‐intensity ultrasonic processor (Scientz, CHN). The lysate was then centrifuged at 12 000 × *g* and 4 °C for 10 min to remove any remaining debris, and the supernatant was collected. The protein concentration was determined by a BCA assay (Thermo Fisher Scientific, USA) according to the manufacturer's instructions.

For digestion, the protein supernatant was reduced with 5 mm dithiothreitol (Sigma–Aldrich, CHN) at 56 °C for 30 min and alkylated with 11 mm iodoacetamide (Sigma–Aldrich, CHN) at RT for 15 min away from light. Next, 100 mm tetraethyl‐ammonium bromide (TEAB, Sigma–Aldrich) was used to dilute the protein sample to a urea concentration less than 2 m. Then, trypsin (Promega, USA) was added at a mass ratio of 1:50 to the protein sample for the first digestion overnight and a 1:100 trypsin‐to‐protein mass ratio for a second 4 h digestion. Finally, the tryptic peptides were analyzed using an LC–MS/MS system (UPLC, nanoElute UHPLC system [Bruker Daltonics]; MS/MS, timsTOF Pro [Bruker Daltonics] mass spectrometry).

### Identification of O‐GlcNAcylated Proteins by LC–MS/MS Analysis

Urea buffer (8 m urea, 100 mm Tris/HCl, pH: 8.5) was used for OEGF‐HUVEC cell lysis and protein extraction. Following quantification with the BCA Protein Assay Kit, the protein sample was digested with 2% trypsin at 37 °C overnight for peptides. The lyophilized peptides were reconstituted in 1.4 mL of precooled IAP Buffer (PTMScan IAP Buffer), and pretreated anti‐GlcNAc‐S/T antibody beads (PTMScan O‐GlcNAc [GlcNAc‐S/T] Motif Kit, CST) were added to enrich for O‐GlcNAcylated peptides. LC–MS/MS analysis was performed on a timsTOF Pro mass spectrometer (Bruker, GER) coupled to nanoElute (Bruker Daltonics). The raw MS data for each sample were combined and searched using Peaks software (BSI, CAN) for identification and quantification. The related parameters and instructions were as follows: peptide mass tolerance, 15.00 ppm; MS/MS tolerance, 0.50 Da; missed cleavage, 2; fixed modification, carbamidomethylation (C); variable modification, acetylation (protein N‐term), oxidation (M), and HexNAcylation (ST).

### SerRS Protein Stability Assay

HUVECs or OEGF cells transfected with SerRS‐His or SerRS‐S101A‐His plasmids were administered 40 µm cycloheximide with or without 50 µm PUGNAc or 50 µm OSMI‐1. After 0, 6, or 12 h of treatment, the cells were scratched and lysed for total protein extraction, which was then subjected to immunoblotting analysis using an anti‐His antibody.

### Immunoprecipitation

HUVECs were transfected with blank vector or vectors expressing SerRS‐His, SerRS‐S101A‐His, SerRS‐S142A‐His, or SerRS‐S146A‐His for 48 h, followed by administration of 50 µm PUGNAc for 24 h. Cells were then harvested and resuspended in precooled immunoprecipitation (IP) lysis buffer (Beyotime, CHN) containing protease inhibitor cocktail (Bimake, CHN). Next, the washed cell lysates were incubated with 2.5 µg immunoglobulin G (IgG) or equal amounts of anti‐His antibody at 4 °C overnight and then supplemented with 30 µL protein A/G magnetic beads (MCE, CHN) at 4 °C for 8 h. Finally, the complexes were eluted and used for immunoblotting analyses.

HUVECs or 293T cells were cotransfected with vectors expressing OGT‐HA and vectors expressing either SerRS‐His, ΔTBD‐His, ΔCD‐His, or ΔUNE‐S‐His. HUVECs, OEGF, or GFKO cells were cotransfected with Ub‐Flag and either SerRS‐His or SerRS‐S101A‐His. HUVECs or 293T cells were cotransfected with vectors expressing importin *α*5‐Flag and either SerRS‐His or SerRS‐MT_NLS_‐His. After 48 h of transfection and combined treatment with 50 µM PUGNAc, 50 µM OSMI‐1, or 20 µM DON for 24 h or lack of treatment, the cells were washed and then resuspended in precooled immunoprecipitation lysis buffer supplemented with 2.5 µg IgG or equal amounts of anti‐His, anti‐Flag, or anti‐HA antibodies at 4 °C overnight, followed by incubation with 30 µL protein A/G magnetic beads at 4 °C for 8 h. Then, the immunoprecipitated complexes were eluted for further immunoblotting analyses.

### sWGA Pull‐Down Assay

Preprocessed cells were washed and lysed in lysis buffer (Beyotime, CHN) containing protease and phosphatase inhibitor cocktails and were further denatured in glycoprotein‐denaturing buffer at 100 °C for 10 min. The cooled lysate was then supplemented with PNGase (NEB, USA) to remove N‐linked glycoproteins. Next, the cell lysate was washed and incubated with succinylated wheat germ agglutinin (sWGA) biotin conjugated beads (Vector Laboratories, USA) at 4 °C overnight. Then, the immunoprecipitated complexes were eluted for further immunoblotting analyses.

### ChIP

Cells were crosslinked with 1% formaldehyde (final concentration) and shaken at RT for 5 min. Then, 2.5 m glycine was added to a final concentration of 125 mm at RT for 5 min with shaking to stop crosslinking. After washing with precooled PBS twice, the cells were scraped into 2 mL lysis buffer (50 mm HEPES, 150 mm NaCl, 1 mm EDTA, 0.1% SDS, 0.1% sodium deoxycholate, 1% Triton X‐100) containing 1X proteinase inhibitors and lysed on ice for 10 min. Next, the lysate was centrifuged at 8000 × *g* and 4 °C for 5 min and washed with PBS. Then, the pellet was sonicated and disaggregated in nuclear lysis buffer (10 mm Tris‐HCl, pH: 8.0, 1 mm EDTA, and 1% SDS) containing 1X protease inhibitor to break the chromatin into fragments and left to stand on ice for 10 min. Sheared chromatin was incubated with IgG, anti‐His, or anti‐Flag antibody (Cell Signaling Technology, CST, USA) bound to protein A/G magnetic beads at 4 °C overnight, followed by elution and reverse cross‐linking at 65 °C overnight. Then, TE buffer (10 mm Tris‐HCl and 1 mm EDTA) was added to the DNA elution buffer, followed by RNase treatment (0.5 mg mL^−1^) at 37  °C for 30 min and proteinase K treatment (0.3 mg mL^−1^) at 51 °C for 1 h. Finally, DNA fragments were isolated and purified by filtration and then quantified by quantitative real‐time PCR (qRT–PCR).

### Luciferase Reporter Assay

293T cells cultured in 96‐well culture plates at a density of 2 × 10^4^ cells/well were cotransfected with VEGFA promoter pGL3‐basic plasmid containing firefly luciferase reporter, an internal control PRL‐TK plasmid, and pcDNA3.1 plasmids containing cDNA encoding certain residues of SerRS or transcription factors at a ratio of 5:1:5. After 6 h of transfection, the transfection medium was replaced with DMEM containing 10% FBS. Following an additional 48 h of transfection, luciferase activity was measured by the Dual‐Luciferase Reporter Assay System (Promega, USA). Renilla luciferase activity was normalized to firefly luciferase activity.

### qRT–PCR Analysis

RNA was extracted using TRIzol reagent (Takara, Japan) according to the manufacturer's instructions. Isolated total RNA was used for reverse transcription with a PrimeScript qRT–PCR kit (Takara, Japan), and a negative control reaction including all reagents except the sample was performed to ensure specificity of the amplification process. qRT–PCR assays were conducted using the SYBR(R) Prime‐Script RT–PCR kit (Takara, Japan) and an ABI 7500 Sequence Detection System (Applied Biosystems, USA). GAPDH was used as an internal control, and the gene expression level was calculated using the Equation 2^–ΔCT^ and was further normalized according to the expression level of the control group. All samples were run in triplicate.

### IB Assay

Total cell protein was obtained using radioimmunoprecipitation lysis buffer (Beyotime, CHN) supplemented with protease inhibitor (Bimake, CHN). Methods for sEV protein extraction and concentration determination were the same as those described above. The extraction of cytoplasmic and purified nuclear proteins was performed using an NE‐PER Nuclear and Cytoplasmic Extraction Reagent Kit (Thermo Scientific, USA) according to the manufacturer's instructions. Immunoblotting analysis was performed as described previously.^[^ ^31^ ^]^ SDS–PAGE gels and PVDF membranes (Millipore, MA, USA) were used for protein separation and blotting, respectively. The protein bands were visualized with enhanced chemiluminescence (Bio–Rad, USA) and quantified using ImageJ software (NIH, USA). Experiments were performed in triplicate. The primary antibodies used in the study are illustrated in Table S2, Supporting Information.

### Enzyme‐Linked Immunosorbent Assay

sEV protein was extracted as described above. A GFAT1 enzyme‐linked immunosorbent assay (ELISA) Kit (Antibodies‐online GmbH, GER) was used to quantify GFAT1 levels in sEVs according to the manufacturer's instructions. Then, 100 µL standard or sample was added to each well, followed by incubation at RT for 2.5 h. Then, 100 µL biotinylated GFAT1 antibody was added to each well and incubated at RT for 1 h. Next, 100 µL prepared streptavidin solution was added to each well and incubated at RT for 45 min, followed by reaction with TMB One‐Step substrate reagent at RT for 30 min. After application of 100 µL stop solution to end the reaction, the optical density was immediately read at 450 nm.

### IF Assay

Bladder tumor and normal tissue slices were stained with anti‐GFAT1 and anti‐CD31 antibodies. TRIM16, GFAT1, and TSG101 staining was performed with anti‐TRIM16, anti‐GFAT1, and anti‐GFP antibodies using UM‐UC‐3 cells transfected with TSG101‐GFP vectors. SerRS and OGT staining was conducted with anti‐His and anti‐OGT antibodies, respectively, using HUVECs transfected with SerRS‐His vectors. SerRS staining was performed with anti‐His antibody using HUVECs transfected with SerRS‐His or SerRS‐MT‐NLS‐His vectors. Goat anti‐rabbit IgG (Alexa Fluor 488 Conjugate), goat anti‐rabbit IgG (Alexa Fluor 647 Conjugate), and goat anti‐mouse IgG (Alexa Fluor 555 Conjugate) secondary antibodies were used for signal visualization. DAPI (CST, USA) was used for nuclear staining. Images were obtained using laser confocal microscopy (Leica Microsystems AG).

### IHC Assay

IHC assays were performed as described previously.^[^ ^103^, ^104^ ^]^ Bladder tumor and normal tissues following formalin fixation and paraffin embedding were processed for sectioning and immunostaining. Serial sections of the tissue were stained with primary antibodies against CD31 (1:200), GFAT1 (1:200), or VEGFA (1:100) and peroxidase‐conjugated anti‐rabbit or anti‐mouse IgG secondary antibody (Thermo Scientific, USA). The signal was visualized by 3,3′‐diaminobenzidine (Thermo Scientific, USA), and slices were scanned using Pannoramic SCAN (3DHISTECH, HUN).

### In Vitro Tube Formation Assay

200 µL of Matrigel (BD, USA, 354230) was coated in each well of a 48‐well plate followed by incubation at 37 °C for 1 h. HUVECs (1 × 10^5^) were resuspended in 100 µL precooled PBS, which was then evenly seeded on the congealed Matrigel. After incubation at 37 °C for 4 h, tube formation was observed using a digital camera system (Olympus, Japan) and further analyzed by ImageJ software (NIH, USA).

### Migration and Invasion Assays

Transwell assays were used to detect the migration and invasion abilities as previously described.^[^ ^31^ ^]^ HUVECs (5 × 10^4^) resuspended in 200 µL serum‐free medium were evenly seeded into a Transwell chamber (Corning, USA) in which the Matrigel was first coated for the invasion assay. The lower compartment of the Transwell chamber was filled with 700 µL of complete medium. After 24 h of migration and 48 h of invasion at 37 °C in a humidified atmosphere of 5% CO_2_, cells were fixed with 4% paraformaldehyde and stained with 0.2% crystal violet. Finally, the migrated and invaded cells were observed using a digital camera system and counted by ImageJ software.

### Statistical Analyses

GraphPad Prism 5 (GraphPad software, USA) and SPSS were used for statistical analyses. Data are presented as the mean ± SD from more than three independent experiments. The statistical significance for comparisons of two groups was detected using unpaired or paired 2‐tailed Student's *t‐*test. One‐way ANOVA followed by Tukey's multiple comparisons test was used for comparisons between multiple groups. Pearson's correlation coefficient was employed to determine linear correlations. The chi‐square test and Fisher's exact probability method were employed to evaluate the differences in clinicopathological characteristics between patients with NMIBC and MIBC. *p* < 0.05 was considered to represent a statistically significant difference. *^/%^ *p* < 0.05, **^/%%^ *p* < 0.01, ***^/%%%^ *p* < 0.001.

## Conflict of Interest

The authors declare no conflict of interest.

## Authors Contribution

Conceptualization: L.XY, G.X., and Z.X. Methodology: L.XY and Z.X. Revision: L.XY, B.XS, and Z.X. Investigation: L.XY, P.X., Z.CL, L.Y., C.G., G.HX, and Z.X. Writing‐original draft: L.XY and P.X. Funding acquisition: H.WY and G.X. Resources: H.WY and G.X. Supervision: Z.X. and G.X.

## Supporting information

Supporting Information

## Data Availability

The data that support the findings of this study are available from the corresponding author upon reasonable request.
