# Supplementary material for: Bladder Cancer‐Derived Small Extracellular Vesicles Promote Tumor Angiogenesis by Inducing HBP‐Related Metabolic Reprogramming and SerRS O‐GlcNAcylation in Endothelial Cells
Source: Adv Sci (Weinh). 2022 Aug 31;9(30):2202993. doi: 10.1002/advs.202202993 (PMC9596856; doi:10.1002/advs.202202993)
Supplement: Supplementary file 1 — Supporting Information [file ADVS-9-2202993-s001.pdf]

ADVANCED SCIENCE
Open Access

Supporting Information

for *Adv. Sci.*, DOI 10.1002/advs.202202993

Bladder Cancer-Derived Small Extracellular Vesicles Promote Tumor Angiogenesis by Inducing HBP-Related Metabolic Reprogramming and SerRS O-GlcNAcylation in Endothelial Cells

Xinyuan Li, Xiang Peng, Chunlin Zhang, Xuesong Bai, Yang Li, Guo Chen, Huixia Guo, Weiyang He, Xiang Zhou and Xin Gou**

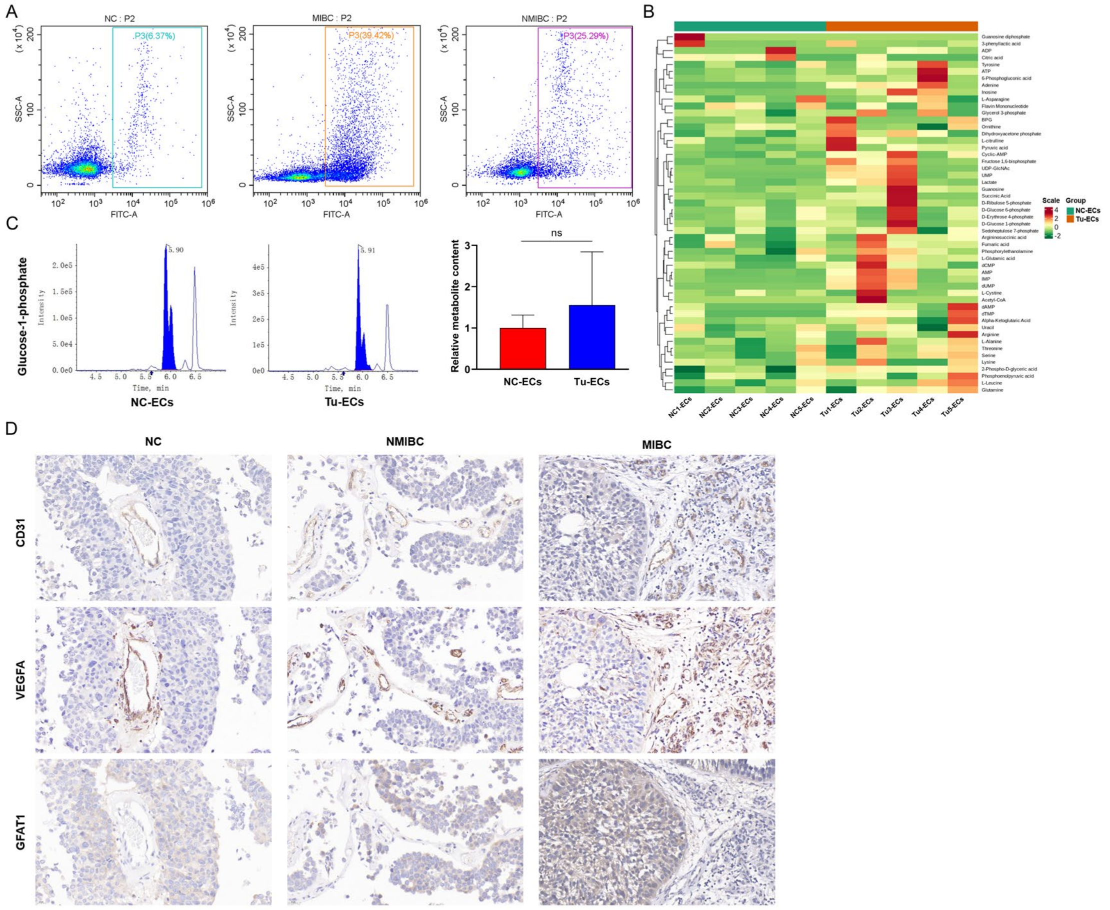

Figure S1. GFAT1 promotes tumour angiogenesis and enhances HBP flux. A The EC percentage was detected in paired Tu and NC of MIBC patients (n = 96) and Tu of NMIBC patients (n = 124) by Flow cytometry. **B** Cluster heat map illustrating the identified glucose metabolism-related metabolites in NC-ECs and Tu-ECs. **C** The relative levels of Glucose-1-phosphate in paired NC-ECs and Tu-ECs (n = 5) (*left*), and quantitative analysis (*right*). ns represents no significant difference between groups. Metabolite content was normalized according to the level in NC-ECs. **D** The indicated proteins in paired Tu and NC of MIBC patients (n = 96) and Tu of NMIBC patients (n = 124) were evaluated by IHC assays of serial sections (scale bar: 20 μm).

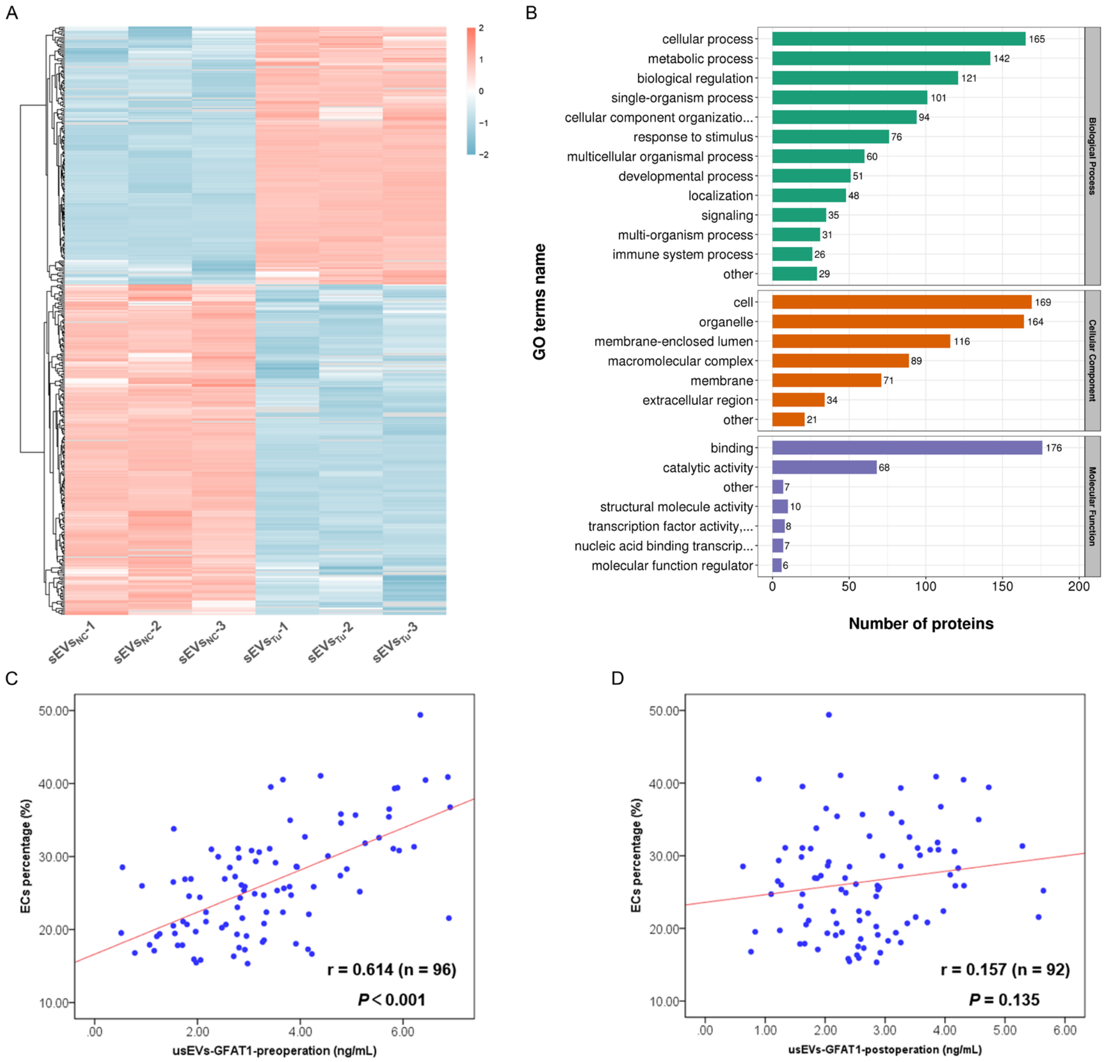

Figure S2. BCa-derived sEVs are associated with metabolism process and angiogenesis. A Cluster heat map illustrating the differentially expressed proteins in $sEVs_{NC}$ (n = 3) and $sEVs_{Tu}$ (n = 3). Proteins with fold variation greater than 2 are shown. **B** Kyoto Encyclopedia of Genes and Genomes (KEGG) analyses of the upregulated proteins in the $sEVs_{Tu}$. **C** Correlation analysis between the GFAT1 concentration in the pre-usEVs and EC percentage in Tu of MIBC patients (n = 96). **D** Correlation analysis between the GFAT1 concentration in the post-usEVs and EC percentage in Tu of MIBC patients (n = 92).

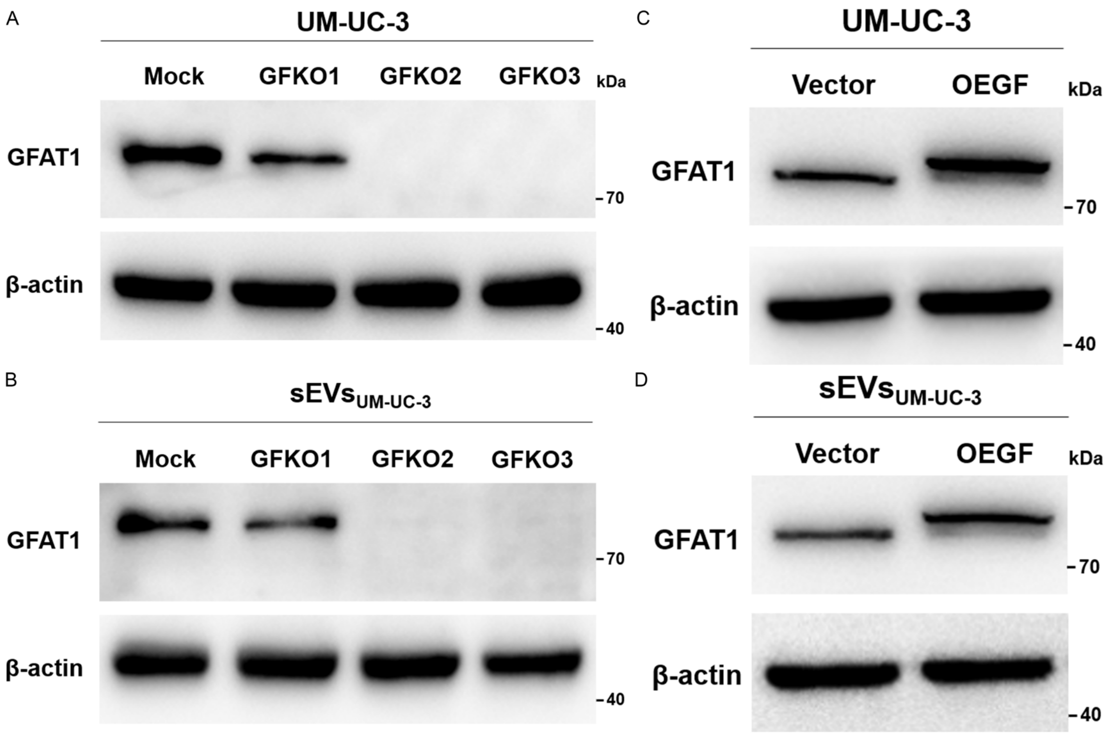

Figure S3. GFAT1 expression in BCa cells directly influences the level of GFAT1 secreted via sEVs. A-B The expression of GFAT1 in GFAT1-knockout UM-UC-3 cells and sEVs derived from the GFAT1-knockout UM-UC-3 cells. **C-D** IB results showing the expression of GFAT1 in the stable overexpression UM-UC-3 cells and sEVs.

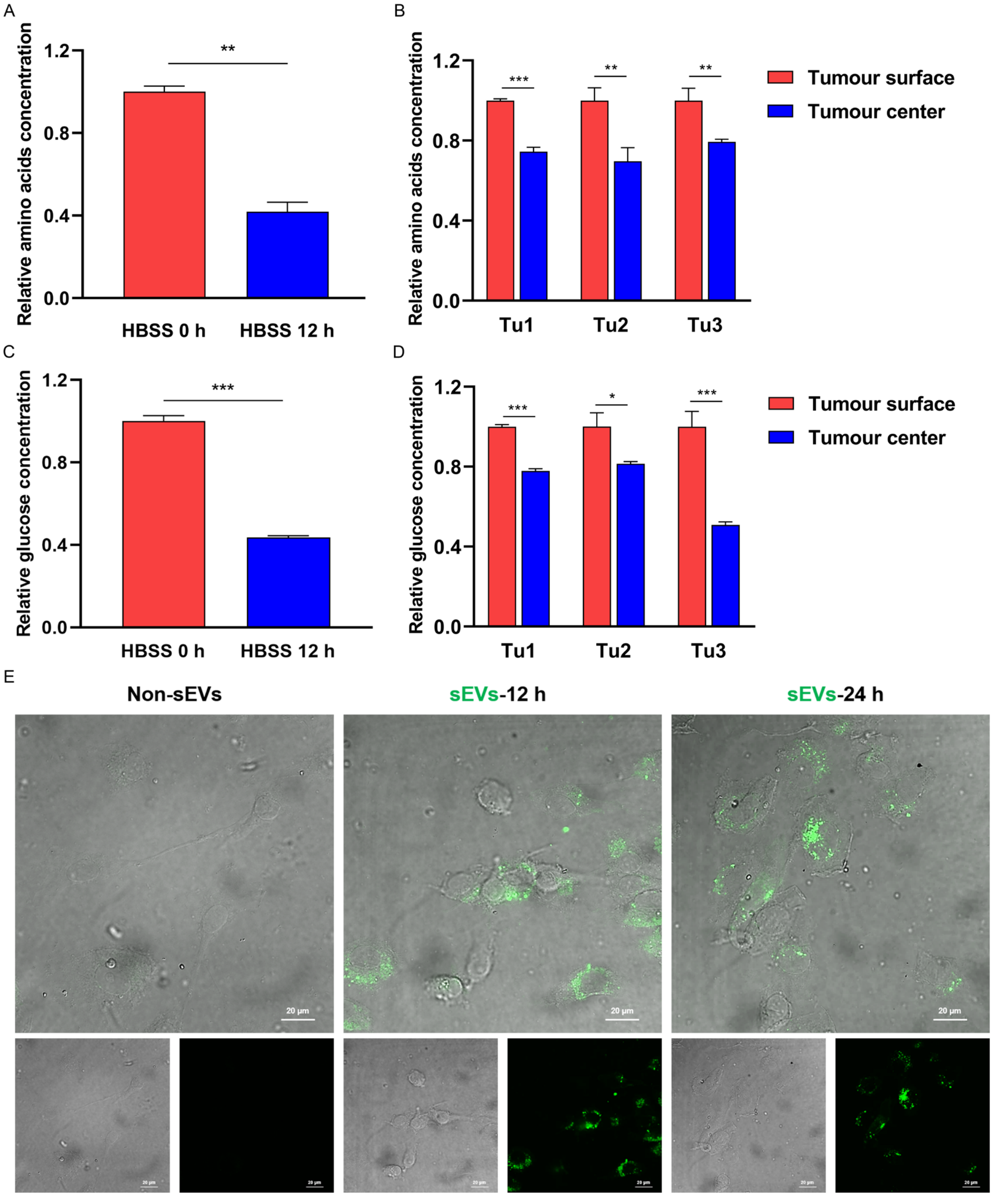

Figure S4. HBSS-induced starvation model in vitro and sEVs-mediated TME model in vitro. **A, C** The relative concentration of amino acids and glucose in UM-UC-3 cells before and after HBSS treatment (12 h). $^{***}P<0.001$, $^{**}P<0.01$. **B, D** The relative concentrations of amino acids and glucose in tissues of bladder tumour surface and center. $^{***}P<0.001$, $^{**}P<0.01$, $^{*}P<0.05$. **E** IF assays recorded the intracellular uptake of the BCa-derived sEVs (labelled with PKH67, green) by HUVECs. Scale bar: 20 μm.

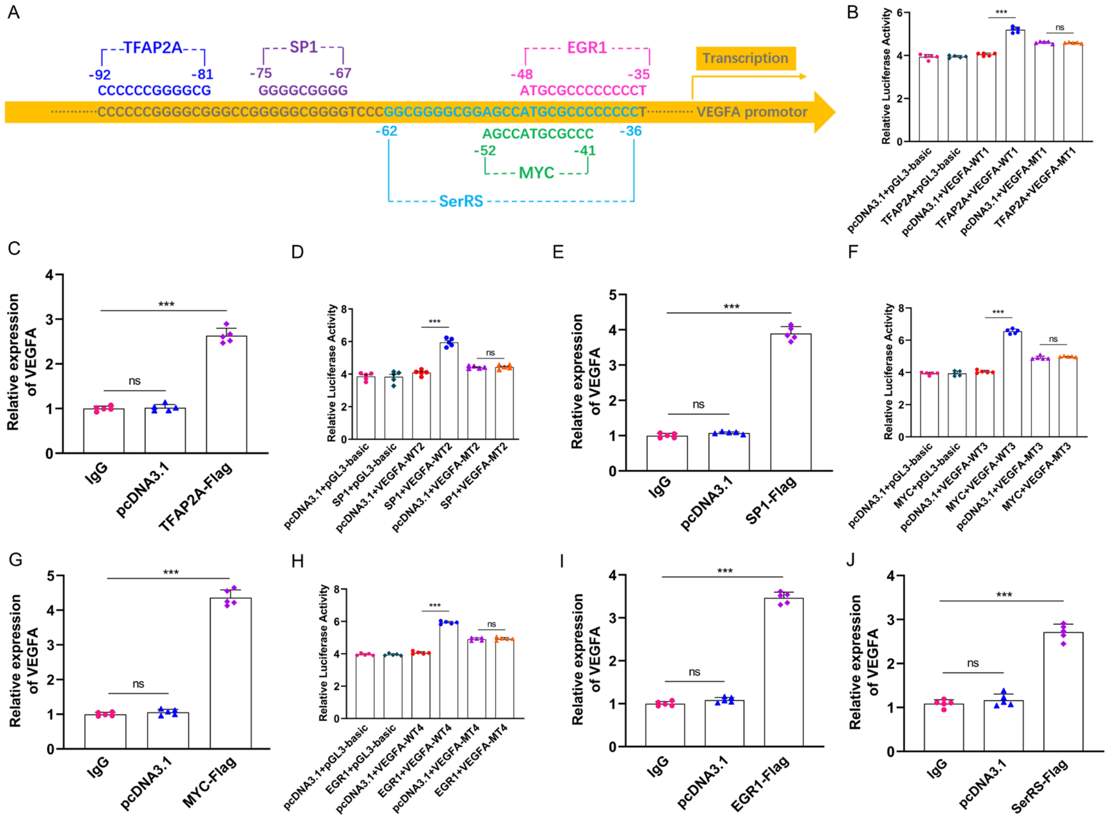

Figure S5. GC-TFs and SerRS can specifically bind to VEGFA proximal promotor. A Schematic representation of the binding sites of TFs on VEGFA proximal promotor, and the site-specific mutants. Luciferase reporter results and ChIP-qPCR results illustrating the binding specificities of TFAP2A (**B-C**), SP1 (**D-E**), MYC (**F-G**) and EGR1 (**H-I**) on the GC-rich region of the VEGFA proximal promoter. **J** ChIP-qPCR assay validated the binding specificity of SerRS on the VEGFA proximal promoter.

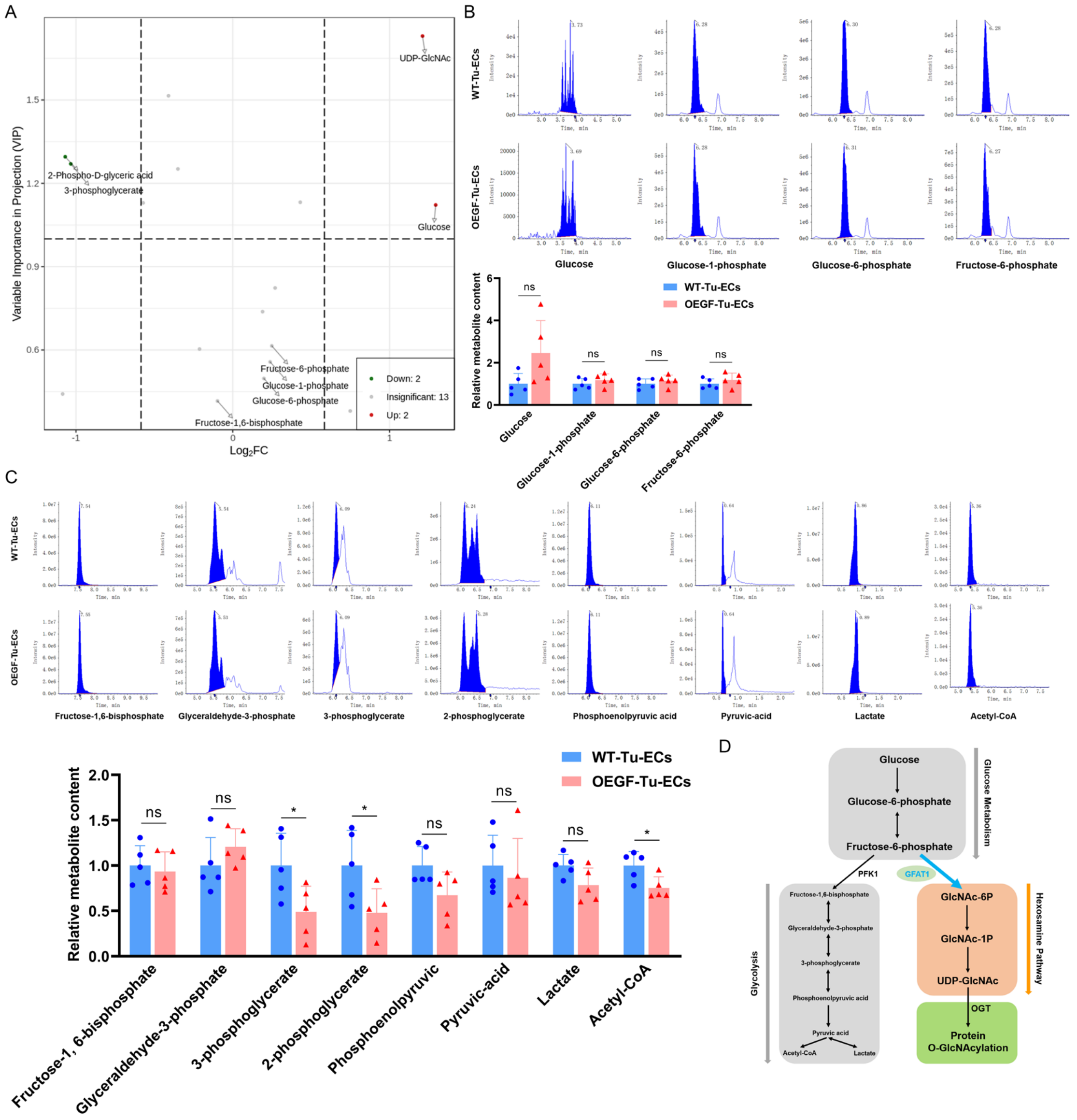

Figure S6. GFAT1 reprograms the glucose metabolic process in Tu-ECs. A Volcano plot showed all detected glucose metabolism-related metabolites in WT-Tu-ECs (n = 5) and OEGF-Tu-ECs (n = 5). The red dots represent the significantly up-regulated metabolites in the Tu-ECs; the green dot represents the remarkably down-regulated metabolites, and the gray dots indicate no significant difference. The levels of some glucose metabolism-related metabolites before the HBP branch (**B**) and in the glycolysis pathway (**C**) in Tu-ECs of WT and OEGF mice (n = 5/group), and quantitative analysis. $^{*}P<0.05$, ns represents no significant difference between groups. **D** Schematic representation of HBP and glycolysis pathway in the glucose metabolism process. Glucose feed into HBP which produces UDP-GlcNAc for O-GlcNAcylation. GFAT1 is the switch and rate-limiting enzyme of HBP.

Table S1: The sgRNA and primer sequences

Item		Sequence
**sgRNA**		
**GFPT1-sgRNA-1**		GCGCCGACACGACTCCCTCG
**GFPT1-sgRNA-2**		TGCCGGAGACACGGCCCGCG
**GFPT1-sgRNA-3**		CGACTCCCTCGGGGATGCGA
**GAL4-sgRNA**		AACGACTAGTTAGGCGTGTA
**siRNA**		
**siR-KPNA1-1**		GCAUGUUGGACGAUAUCUAAU
**siR-KPNA1-2**		GGUUUGUGGAGUUCCUCAAAC
**Primer**		
**VEGFA-Primer 1**	Forward	TTGCCTTGCTGCTCTACCTCCA
	Reverse	GATGGCAGTAGCTGCGCTGATA
**VEGFA-Primer 2**	Forward	TCAGGCTGTGAACCTTGGTG
	Reverse	CCGCTACCAGCCGACTTTTA
**VEGFA-Primer 3**	Forward	CGTGTGGAAGGGCTGAGG
	Reverse	GCTGACCGGTCCACCTAAC
**VEGFA-Primer 3**	Forward	TGGAAGGGCTGAGGCTCG
	Reverse	GCTGACCGGTCCACCTAAC
**GAPDH**	Forward	CCTTCCGTGTCCCCACT
	Reverse	GCCTGCTTCACCCACCTTC

Table S2: VEGFA wild-type promotor sequences and mutants

Name	Sequence (5'-3')
**VEGFA-WT**	GGTACCTTCCCCTTCATTGCGGCGGGCTGCGGGCCAGGCTTCACTGAGCGTCC<br>GCAGAGCCCGGGCCCGAGCCGCGTGTGGAAGGGCTGAGGCTCGCCTGTCCCC<br>GCCCCCCGGGGCGGGCCGGGGGCGGGGTCCCGGCGGGGCGGAGCCATGCGCC<br>CCCCCCTTTTTTTTTTTAAAAGTCGGCTGGTAGCGGGGAGGATCTCGAG
**VEGFA-MT1**	GGTACCTTCCCCTTCATTGCGGCGGGCTGCGGGCCAGGCTTCACTGAGCGTCC<br>GCAGAGCCCGGGCCCGAGCCGCGTGTGGAAGGGCTGAGGCTCGCCTGTCCCC<br>GTTTTTTAAAATAGGCCGGGGGCGGGGTCCCGGCGGGGCGGAGCCATGCGCC<br>CCCCCCTTTTTTTTTTTAAAAGTCGGCTGGTAGCGGGGAGGATCTCGAG
**VEGFA-MT2**	GGTACCTTCCCCTTCATTGCGGCGGGCTGCGGGCCAGGCTTCACTGAGCGTCC<br>GCAGAGCCCGGGCCCGAGCCGCGTGTGGAAGGGCTGAGGCTCGCCTGTCCCC<br>GCCCCCCGGGGCGGGCCGAAAATAAAATCCCGGCGGGGCGGAGCCATGCGCC<br>CCCCCCTTTTTTTTTTTAAAAGTCGGCTGGTAGCGGGGAGGATCTCGAG
**VEGFA-MT3**	GGTACCTTCCCCTTCATTGCGGCGGGCTGCGGGCCAGGCTTCACTGAGCGTCC<br>GCAGAGCCCGGGCCCGAGCCGCGTGTGGAAGGGCTGAGGCTCGCCTGTCCCC<br>GCCCCCCGGGGCGGGCCGGGGGCGGGGTCCCGGCGGGGCGGGATTGCATATT<br>TCCCCCTTTTTTTTTTTAAAAGTCGGCTGGTAGCGGGGAGGATCTCGAG
**VEGFA-MT4**	GGTACCTTCCCCTTCATTGCGGCGGGCTGCGGGCCAGGCTTCACTGAGCGTCC<br>GCAGAGCCCGGGCCCGAGCCGCGTGTGGAAGGGCTGAGGCTCGCCTGTCCCC<br>GCCCCCCGGGGCGGGCCGGGGGCGGGGTCCCGGCGGGGCGGAGCCGCATATT<br>TTTTTTCTTTTTTTTTAAAAGTCGGCTGGTAGCGGGGAGGATCTCGAG

Note: The mutants of binding sites are marked red. WT: wide-type; MT: mutant.

Table S3. Antibodies used for IB, IP, ChIP, IHC, IF, FCM and ELISA

**Western blotting**			
**Antibodies**	**Source**	**Antibody dilution**	**Identifier**
Rabbit anti-GFPT1	Abcam	1:1000	Cat#ab125069
Mouse anti-O-GlcNAc	Abcam	1:1000	Cat#ab2739
Rabbit anti- SERS	Abcam	1:1000	Cat#ab154825
Rabbit anti- OGT	Abcam	1:1000	Cat#ab96718
Rabbit anti-CD9	Abcam	1:1000	Cat# ab236630
Mouse anti-TSG101	Abcam	1:1000	Cat# ab83
Rabbit anti-His	Cell Signaling Technology	1:1000	Cat#ab12698
Rabbit anti-HA	Cell Signaling Technology	1:1000	Cat#ab3724
Rabbit anti-Flag	Cell Signaling Technology	1:1000	Cat#ab14793
Rabbit anti-Calnexin	Cell Signaling Technology	1:1000	Cat# 2679
Rabbit anti-p-GFAT1 (Ser243)	Immuno-Biological Laboratories	1:500	Cat# 28123
Mouse anti-β-actin	Proteintech	1:5000	Cat# 66009-1-Ig
Rabbit anti-PCNA	Proteintech	1:5000	Cat# 10205-2-AP
Mouse anti-GAPDH	Proteintech	1:5000	Cat# 60004-1-Ig
Rabbit anti-VEGFA	Proteintech	1:1000	Cat# 19003-1-AP
Rabbit anti-KPNA	Proteintech	1:1000	Cat# 18137-1-AP
Rabbit anti-UMOD	R&D Systems	1:1000	Cat# MAB5175
**IP**			
Mouse anti-DYKDDDDK-Tag	Abmart	1:100	Cat# M20008
Mouse anti-His-Tag	Abmart	1:100	Cat# M20001
Mouse anti-HA-Tag	Abmart	1:100	Cat# M20003
Rabbit anti-OGT	Abcam	1:100	Cat# ab96718
Rabbit anti- SERS	Abcam	1:100	Cat# ab183025
sWGA-conjugated agarose beads	Vector Laboratories	1:10	Cat# AL-1023S
Protein A/G magnetic beads	MCE		Cat# HY-K0202
**ChIP**			
Rabbit anti-His	Cell Signaling Technology	1:50	Cat#ab12698
Rabbit anti-HA	Cell Signaling Technology	1:50	Cat#ab3724
**IHC**			
Rabbit anti-GFPT1	Abcam	1:200	Cat#ab125069
Mouse anti-O-GlcNAc	Abcam	1:100	Cat#ab2739
Rabbit anti-CD31	Abcam	1:200	Cat# ab28364
Rabbit anti-VEGFA	Proteintech	1:100	Cat# 19003-1-AP
**IF**			
Rabbit anti-GFPT1	Abcam	1:100	Cat#ab125069
Mouse anti-O-GlcNAc	Abcam	1:100	Cat#ab2739
Rabbit anti- OGT	Abcam	1:1000	Cat# ab96718
Rabbit anti-VEGFA	Proteintech	1:100	Cat# 19003-1-AP
Rabbit anti-TRIM16	Proteintech	1:100	Cat# 24403-1-AP

DAPI	Cell Signaling Technology	1:100	Cat# 4083
Goat anti-Rabbit IgG (Alexa Fluor® 488 Conjugate)	Cell Signaling Technology	1:500	Cat# 4412
Goat Anti-Mouse IgG (Alexa Fluor® 555 Conjugate)	Cell Signaling Technology	1:500	Cat# 4409
Goat Anti- Rabbit IgG (Alexa Fluor® 647 Conjugate)	Cell Signaling Technology	1:500	Cat# 4414
**FCM**			
FITC anti-human CD31	Biolegend	1:100	Cat#303104
FITC anti-mouse CD31 Antibody	Biolegend	1:100	Cat#102406
Fc Receptor Blocking Solution	Biolegend		Cat# 422301
**CD31 and ELISA Kit**			
CD31 Microbead Kit, human	Miltenyi biotec	-	Cat# 130-091-935
CD31 Microbead Kit, mouse	Miltenyi biotec	-	Cat# 130-097-418
GFPT1 ELISA Kit	Antibodies-online GmbH	-	Cat# ABIN6951707

Table S4. Chemicals, critical commercial assays and experimental models

**Chemicals**		
**RESOURCE**	**SOURCE**	**IDENTIFIER**
2× Taq PCR Green Mix	Takara	RR820A
RNAiso Plus	Takara	9108
6-Diazo-5-oxo-L-nor-Leucine	MCE	HY-108357
PNGaseF (Glycerol-free), Recombinant	New england biolabs	P0709S
PUGNAc	Sigma–Aldrich	A7229
OSMI-1	Sigma–Aldrich	SML1621
Thiamet G	Sigma–Aldrich	SML0244
MG132	Sigma–Aldrich	M7449
CHX	Sigma–Aldrich	5087390001
PKH67	Sigma–Aldrich	MINI67
Lipofectamine 2000	Invitrogen	11668019
Lipofectamine 3000	Invitrogen	L3000015
N-butyl-N-4-hydroxybutyl nitrosamine	TCI	B0938
**Critical Commercial Assays**		
BCA protein assay Kit	Thermo Fisher Scientific	23227
Nuclear and Cytoplasmic Protein Extraction Kit	Thermo Fisher Scientific	78833
Hank's balanced salt solution	Thermo Fisher Scientific	14025076
DAB kit	Thermo Fisher Scientific	34002
Cell lysis buffer for Western and IP	Beyotime	P0013
RIPA	Beyotime	P0013B
PMSF	Beyotime	ST506
4% Paraformaldehyde	Beyotime	P0099
Penicillin-Streptomycin	Beyotime	C0223
Dual-Luciferase® Reporter Assay System	Promega	E1910
PVDF membranes	Millipore	ISEQ00010
PrimeScript® RT reagent Kit	Takara	RR047A
Protease Inhibitor Cocktail	Bimake	B14001
RPMI 1640 medium	Gibco	C11875500BT
Dulbecco’s modified Eagle’s medium	Gibco	C11995500BT
Foetal bovine serum	BioInd	04-001-1A
Bovine serum albumin	Sigma–Aldrich	9048-46-8
Red blood cell lysis buffer	Solarbio	R1010
EDTA	Solarbio	E1170
70 mm Cell-Strainer	Becton,Dickinson and Company	352350
Gadopentetic acid	MCE	HY-107353
Matrigel	BD	354230
Transwell chamber	Corning	3413

Micro amino acid content assay kit	Solarbio	BC1575
Glucose content assay kit	Solarbio	BC2505
**Cell Lines**		
UM-UC-3	Cell Bank of the Chinese Academy of Sciences	TCHu217
293T	Cell Bank of the Chinese Academy of Sciences	GNHu17
Human umbilical vein endothelial cells	American Type Culture Collection	PCS-100-010
**Experimental Models**		
C57BL/6-Gfat1$^{+/-}$ mice	SHANGHAI MODEL ORGANISMS	-
BALB/c nude mice	AAALCA-accredited SPF Biotechnology	-
